# Reallocating Time Between 24-h Movement Behaviors for Obesity Management Across the Lifespan: A Pooled Data Meta-Analysis of More Than 9800 Participants from Seven Countries

**DOI:** 10.1007/s40279-024-02148-4

**Published:** 2024-12-21

**Authors:** Aleš Gába, Timothy B. Hartwig, Paulína Jašková, Taren Sanders, Jan Dygrýn, Ondřej Vencálek, Devan Antczak, James Conigrave, Phillip Parker, Borja del Pozo Cruz, Stuart J. Fairclough, Shona Halson, Karel Hron, Michael Noetel, Manuel Ávila-García, Veronica Cabanas-Sánchez, Iván Cavero-Redondo, Rachel G. Curtis, Bruno G. G. da Costa, Jesus del Pozo-Cruz, Antonio García-Hermoso, Angus A. Leahy, David R. Lubans, Carol A. Maher, David Martínez-Gómez, Kim Meredith-Jones, Andrés Redondo-Tébar, Séverine Sabia, Kelly S. Silva, Paula Skidmore, Emilio Villa-González, Manasa S. Yerramalla, Chris Lonsdale

**Affiliations:** 1 Faculty of Physical Culture, Palacký University Olomouc, tř. Míru 117, 771 11 Olomouc, Czech Republic; 2 Australian Catholic University, Strathfield, NSW Australia; 3 Australian Catholic University, North Sydney, NSW Australia; 4 Faculty of Science, Palacký University Olomouc, 17. listopadu 12, 779 00 Olomouc, Czech Republic; 5 University of Wollongong, Wollongong, NSW Australia; 6 La Trobe University, Melbourne, VIC Australia; 7 Faculty of Medicine, Health, and Sports, Department of Sport Sciences, Universidad Europea de Madrid, Villaviciosa de Odón, Madrid, Spain; 8 Edge Hill University, Ormskirk, UK; 9 Australian Catholic University, Banyo, QLD Australia; 10 The University of Queensland, St Lucia, QLD Australia; 11 “La Inmaculada” Teacher Training Centre, University of Granada, 18013 Granada, Spain; 12 Faculty of Sport Sciences, University Isabel I, 09003 Burgos, Spain; 13 Department of Preventive Medicine and Public Health, School of Medicine, Universidad Autonoma de Madrid, Madrid, Spain; 14 CIBER of Epidemiology and Public Health, Madrid, Spain; 15 IMDEA-Food Institute, CEI UAM+CSIC, Madrid, Spain; 16 Universidad de Castilla-La Mancha, Cuenca, Spain; 17 University of South Australia, Adelaide, SA Australia; 18 Department of Kinesiology and Physical Education, McGill University, Montreal, QC Canada; 19 Universidad de Sevilla, Seville, Spain; 20 Navarrabiomed, Hospital Universitario de Navarra (HUN), Universidad Pública de Navarra (UPNA), IdiSNA, Navarra, Spain; 21 The University of Newcastle, Callaghan, NSW Australia; 22 Centre for Active Living and Learning, College of Human and Social Futures, University of Newcastle, Callaghan, NSW Australia; 23 Hunter Medical Research Institute, New Lambton Heights, NSW Australia; 24 Faculty of Sport and Health Sciences, University of Jyväskylä, Jyväskylä, Finland; 25 University of Otago, Dunedin, New Zealand; 26 Université Paris Cité, Inserm U1153 EpiAgeing, Paris, France; 27 Universidade Federal de Santa Catarina, Florianopolis, Brazil; 28 University of Otago, Christchurch, New Zealand; 29 University of Granada, Granada, Spain; 30 Université Paris Cité, Inserm, Paris, France

## Abstract

**Background:**

The distribution of time across physical activity, sedentary behaviors, and sleep appears to be essential for the management of obesity. However, the impact of reallocating time among these behaviors, collectively known as 24-h movement behaviors, remains underexplored.

**Objective:**

This study examines the theoretical effects of reallocating time between 24-h movement behaviors on obesity indicators across different age groups.

**Methods:**

We performed a pooled data meta-analysis of 9818 participants from 11 observational and experimental studies. To estimate the time spent in movement behaviors, we reprocessed and harmonized individual-level raw accelerometer-derived data. Isotemporal substitution models estimated theoretical changes in body mass index (BMI) and waist circumference (WC) associated with time reallocation between movement behaviors. We performed the analysis separately for children, adolescents, adults, and older adults.

**Results:**

Even minor reallocations of 10 min led to significant changes in obesity indicators, with pronounced effects observed when 30 min were reallocated. The most substantial adverse effects on BMI and WC occurred when moderate-to-vigorous physical activity (MVPA) was reallocated to other movement behaviors. For 30-min reallocations, the largest increase in BMI (or BMI *z*-score for children) occurred when MVPA was reallocated to light-intensity physical activity (LPA) in children (0.26 units, 95% confidence interval [CI] 0.15, 0.37) and to sedentary behavior (SB) in adults (0.72 kg/m^2^, 95% CI 0.47, 0.96) and older adults (0.73 kg/m^2^, 95% CI 0.59, 0.87). The largest increase in WC was observed when MVPA was substituted with LPA in adults (2.66 cm, 95% CI 1.42, 3.90) and with SB in older adults (2.43 cm, 95% CI 2.07, 2.79). Conversely, the highest magnitude of the decrease in obesity indicators was observed when SB was substituted with MVPA. Specifically, substituting 30 min of SB with MVPA was associated with a decrease in BMI *z*-score by − 0.15 units (95% CI − 0.21, − 0.10) in children and lower BMI by − 0.56 kg/m^2^ (95% CI − 0.74, − 0.39) in adults and by − 0.52 kg/m^2^ (95% CI − 0.61, − 0.43) in older adults. Reallocating time away from sleep and LPA showed several significant changes but lacked a consistent pattern. While the predicted changes in obesity indicators were generally consistent across age groups, inconsistent findings were observed in adolescents, particularly for reallocations between MVPA and other behaviors.

**Conclusions:**

This investigation emphasizes the crucial role of MVPA in mitigating obesity risk across the lifespan, and the benefit of substituting SB with low-intensity movement behaviors. The distinct patterns observed in adolescents suggest a need for age-specific lifestyle interventions to effectively address obesity. Emphasizing manageable shifts, such as 10-min reallocations, could have significant public health implications, promoting sustainable lifestyle changes that accommodate individuals with diverse needs, including those with severe obesity.

**Supplementary Information:**

The online version contains supplementary material available at 10.1007/s40279-024-02148-4.

## Key Points

**Table:** 

Our findings reinforce the importance of moderate-to-vigorous physical activity in the management of obesity and provide novel evidence that benefits can be derived from the substitution of sedentary time, even with lighter-intensity activities.
Positive effects on obesity may be possible by targeting modest lifestyle changes that are feasible and sustainable for a broad range of individuals, including those with severe obesity, low physical capabilities, and other associated health conditions.
Although our findings were generally consistent across age groups, adolescents may require age-specific interventions.

## Introduction

Despite encouraging evidence of a plateau in obesity prevalence in high-income countries, the global trend continues to rise, driven by a sharp increase in emerging countries [1, 2]. According to a recent projection of the World Obesity Federation [2], the global population of those living with obesity is expected to exceed 2 billion by 2035, corresponding to an increase from 14 to 24% within 15 years. The cost of obesity and related diseases is expected to rise dramatically. If current trends persist, the economic impact of overweight and obesity will surpass 3% of global gross domestic product in the coming decades and lower resourced countries will bear the largest economic burden [3]. Obesity, therefore, is a critical global health challenge that requires immediate policy actions to mitigate its impact on global health and development.

Addressing obesity is challenging owing to its multifactorial nature. Although modern approaches to managing obesity recognize many genetic and environmental drivers [4, 5], lifestyle changes remain foundational to any weight loss and maintenance efforts [6]. Regular physical activity is considered an essential component of lifestyle change, complementing the benefits of dietary change [7] and improving the efficacy of modern pharmacological therapy [8]. Physical activity is also effective as a stand-alone intervention [9]. However, achieving long-term benefits from lifestyle interventions can be challenging as various barriers hinder engagement with regular physical activity, especially among those living with chronic conditions [10]. Substituting prolonged sedentary behavior (SB) with light-intensity physical activity (LPA) may help counter these issues and is advised as an initial step to improve adherence and facilitate a progressive transition to moderate-to-vigorous physical activity (MVPA) [11]. Sleep is also crucial for obesity management [12, 13] and should be integrated into multicomponent intervention strategies. Thus, a holistic approach to addressing obesity that encompasses all daily movement behaviors, collectively referred to as 24-h movement behaviors (i.e., sleep, SB, and physical activity of different intensities), has the potential to enhance the effectiveness and sustainability of obesity intervention efforts [14].

A substantial amount of research highlights the potential to reduce the risk of obesity through the reallocation of time between 24-h movement behaviors [15, 16]. A recent scoping review [17] indicates that the reallocation of time from any other behavior to MVPA is associated with the greatest improvements in obesity indicators. Additional benefits can be achieved by reallocating time to sleep and LPA. The review also revealed an inconsistency in the findings depending on age, highlighting the need to expand our understanding of how the reallocation of time between movement behaviors influences obesity indicators at different stages of life. Studying the interaction of movement behaviors across the lifespan is vital for developing lifestyle interventions tailored for specific age groups [18]. However, it is challenging to draw general recommendations based on previous research. This is mainly because most previous studies have concentrated on specific age groups, and often have small sample sizes with limited geographical representation. Additionally, there is considerable variation in methodologies used for processing device-measured data on 24-h movement behaviors, further complicating the derivation of guidelines. To help bridge this evidence gap, we designed the present study to pool harmonized individual participant data from 11 geographically diverse observational and experimental studies of varied cohorts, with high-resolution accelerometer-derived data on 24-h movement behaviors. We employed the compositional isotemporal substitution approach [19] to estimate theoretical changes in obesity indicators associated with time reallocations between sleep, SB, LPA, and MVPA across different age groups.

## Methods

This study is a secondary analysis of an individual participant data study that aimed to examine the relationships between sleep and physical activity across the lifespan. The current analysis adheres to the original protocol [20] in most methodological aspects, and any deviations from the original protocol are described in this section. The Preferred Reporting Items for a Systematic Review and Meta-analysis of Individual Participant Data (PRISMA-IPD) guidelines for meta-analysis of individual participant data were followed [21].

### Search Strategy and Inclusion Criteria

A multi-level search strategy was employed to identify potentially relevant studies. This involved the following steps: (1) searching for relevant studies included in the reference list of the most recent systematic reviews covering the objective of the primary analysis; (2) mapping the literature using the relevant studies from Step 1 as “seed studies”; (3) searching for unpublished research and gray literature in the clinical trials registers (International Clinical Trials Registry Platform [ICTRP], International Standard Randomised Controlled Trial Number [ISRCTN], and Australian New Zealand Clinical Trials Registry [ANZCTR]) and the Open Science Framework database; (4) identifying accelerometry consortiums; and (5) directly contacting researchers to identify relevant data sources. Studies were eligible for inclusion if they provided data on 24-h movement behaviors (i.e., sleep, SB, LPA, and MVPA) derived from research-grade accelerometers. Raw accelerometer data with a sampling frequency of 30–100 Hz had to be collected using a 24-h wear time protocol over at least 4 days, including at least 1 weekend day. To be included, the study sample size had to be more than 400 participants older than 4 years. All studies included in the pooled data meta-analysis had to have been approved by their local institutional ethics committees, and all participants had to have provided written informed consent.

### Individual Data Processing

After eligible studies were identified, the corresponding authors were invited to contribute their data to this study. For experimental studies, only baseline data were requested to maintain consistency with the observational studies. As the present study was conducted as a meta-analysis of individual participant data, authors who agreed were asked to provide de-identified individual raw accelerometer 24-h data and additional individual and study-level data via a secure file-sharing system. Raw accelerometer data were reprocessed for harmonization either by researchers at the lead university or by contributing authors depending on data sharing agreement requirements for each contributing research group. After submitting the research protocol [20] together with letters of agreement from contributing authors, the Human Research Ethics Committee of the Australian Catholic University (NSW, Australia) approved the study under registration number 2020-143N.

The raw data from the wrist-worn accelerometers were reprocessed using the sleepIPD R package version 0.2.2 [22]. This package ensures standardized processing of accelerometer files and offers a suite of auxiliary scripts to streamline metadata generation. The code of sleepIPD is built upon the GGIR R package, the most widely used approach for generating 24-h movement behavior outcomes from raw multi-day accelerometer data [23]. SleepIPD ensured that the same version of GGIR (version 2.5–1) was used on all accelerometer data, to minimize the risk of changes to GGIR impacting the results. This version of GGIR is capable of processing accelerometer data from a wide range of formats (e.g.,.csv or.bin), which the various accelerometer brands use.

GGIR’s algorithms were employed for sleep period detection [24]. We used Euclidean Norm Minus One thresholds [25–27] reflecting the age of the participants and the device brand to estimate the amount of time spent on waking behaviors (i.e., SB, LPA, and MVPA). The specific age- and device-specific thresholds are given in the sleepIPD documentation [22]. To obtain average daily values for the time spent in each of these behaviors, we used the “waking up to waking up the next day” approach, which means that a sleep period is not split by midnight and that the duration of days vary. Only participants with a minimum of 3 valid weekdays and 1 valid weekend day were included in the analysis. A day was considered valid if it had an average wear time in the range of 540–1080 min during wake time, a sleep duration in the range of 320–900 min, and a combined total of wake time and sleep of at least 16 h.

### Obesity Indicators

Body mass index was used as a measure of overall obesity and was calculated using height and weight that were standardized to centimeters and kilograms, respectively. The World Health Organization body mass index *z*-score (BMIz) was calculated for children and adolescents to account for sex- and age-related differences in height and weight occurring during childhood and adolescence. We used waist circumference as an indicator of abdominal obesity. This indicator was available for all age groups except adolescents.

### Statistical Analysis

The analysis was conducted using the R software (version 4.3.1) with compositions, nlme, robCompositions, robustbase, and VIM packages. A *p*-value of less than 0.05 was considered statistically significant in all the analyses. To investigate the effect of time reallocations between movement behaviors on obesity indicators across the lifespan, the analysis was carried out separately for children (aged 5–12 years), adolescents (aged 13–17 years), adults (aged 18–64 years), and older adults (aged 65 years and older).

Multi-level multivariate regression models were used to investigate associations between a four-part time-use composition (consisting of the time spent in sleep, SB, LPA, and MVPA) and obesity indicators. All regression models were mixed models with random intercepts for the study identification number to account for bias from unmeasured demographic and methodological deviations between the included studies. An isometric log-ratio transformation of a time-use composition was carried out to map the 24-h movement behavior data into the set of pivot coordinates (i.e., ilr_1_, ilr_2_, and ilr_3_). We built four models, each allowing one movement behavior to be considered relative to the remaining models with the order of components rearranged to express each behavior as ilr_1_. The first coordinate (ilr_1_) reflects the dominance of a given behavior over the remaining behaviors within the time-use composition. Therefore, the final model aggregates the first pivot coordinates from individual models. The detailed description of this approach is available elsewhere [19]. Regression models were adjusted for sex, age, and socioeconomic status for all age groups. The identification of confounding variables and the description of the sensitivity analysis are detailed in Tables S1 and S2 of the Electronic Supplementary Material (ESM).

A compositional isotemporal substitution model [19] was developed to investigate the theoretical changes in obesity indicators for reallocation of time between sleep, SB, LPA, and MVPA. To ensure that predicted values were comparable between age groups, the reallocations were based on the regression models with an identical set of covariates. One-for-one reallocations were used to estimate the changes in obesity indicators associated with time reallocations between two components of the time-use composition (e.g., from SB to MVPA and vice versa). This approach adequately reflects the compositional nature of time-use data, is the most frequently used in previous research [17], and is the most suitable for public health messaging. The mean time-use composition (i.e., compositional mean) was used as the starting point for prediction purposes with 10-min increments up to 60 min/day. The predicted changes were considered significant when the 95% confidence interval (CI) did not include zero.

## Results

Among the studies that consented to contribute individual participant data for the primary study, 11 studies [28–38] agreed to share data for this secondary analysis (Table 1). The included studies were conducted in the European region (*n* = 5), the Western Pacific region (*n* = 4), and the region of the Americas (*n* = 2). Most studies were representative of countries with a very high human development index (*n* = 10) except for one that represented a country with a high human development index (Brazil). Data on BMI were available from all included studies and five studies provided data on waist circumference. Most studies were cross-sectional and used GENEActiv accelerometers for analyzing 24-h movement behaviors over a minimum of 7 days.

**Table 1:** Characteristics of studies included in the analysis

Study name^a^	Country	Study design	Year of data collection	No. of participants (% female, % included in the analytic sample)	Age groups	Obesity indicators	Accelerometer type (wear location, sampling interval, wear days)
Active-Start study [28]	Chile	Cross-sectional	2018	149 (39%, 74%)	Children	BMI	GENEActiv (NDW, 87.5 Hz, 7)
Active Team study [29]	Australia	RCT	2017–18	371 (73%, 52%)	Adults, older adults	BMI^b^	GENEActiv (NDW, 50 Hz, 7)
Burn 2 Learn study [30]	Australia	RCT	2018–19	460 (47%, 41%)	Adolescents, adults	BMI	ActiGraph GT9X (NDW, 30 Hz, 7)
iPLAY study [31]	Australia	RCT	2016–17	1097 (50%, 72%)	Children	BMI	GENEActiv (NDW, 87.5 Hz, 8)
MOVI-da10! study [32]	Spain	RCT	2017–18	196 (48%, 78%)	Children	BMI, WC	GENEActiv (NDW, 30 Hz, 7)
PEDALS study 5 [33]	New Zealand	Cross-sectional	2015	699 (61%, 81%)	Children, adults	BMI, WC	ActiGraph GT3X + (NDW, 30 Hz, 8)
PREVIENE study [34]	Spain	RCT	2017	361 (47%, 90%)	Children	BMI, WC	ActiGraph wGT3X-BT + (NDW, 90 Hz, 7)
Seniors-ENRICA-2 study [35]	Spain	Cohort	2015–17	2452 (53%, 88%)	Older adults	BMI, WC	ActiGraph GT9X (NDW, 100 Hz, 7)
Study 1 [36]	Brazil	Cross-sectional	2019	795 (50%, 76%)	Adolescents, adults	BMI	ActiGraph GT3X + (NDW, 30 Hz, 7)
Study 2 [37]	Czechia	Cross-sectional	2018–22^c^	1231 (52%, 68%)	Children, adolescents, Adults	BMI	ActiGraph GT3X + and GT9X (NDW, 100 Hz, 7)
Whitehall 2 study [38]	UK	Cross-sectional	2012–13	4202 (26%, 93%)^d^	Adults, older Adults	BMI, WC	GENEActiv (NDW, 87.5 Hz, 9)

*BMI* body mass index, *NDW* non-dominant wrist, *RCT* randomized controlled trial, *UK* United Kingdom,* WC* waist circumference

^a^If available a reference to the study design is presented

^b^Self-reported data

^c^Data were collected before (2018–19) and after (2022) the coronavirus disease 2019 pandemic

^d^National sample

The total sample included 12,013 participants from which 2195 were excluded (see Fig. S1 of the ESM for reasons), mostly because participants did not meet wear time criteria or had highly suspicious activity data for movement behaviors (i.e., > 3 standard deviations). Thus, the final analytic sample included 9818 participants of which 21% were children, 13% were adolescents, 15% were adults, and 51% were older adults. In the final sample, there were 59,732 valid accelerometer-derived person-days. The mean values of average acceleration indicated an age-related decline in the volume of activity, from 79.2 ± 23.3 m*g* in children to 30.9 ± 9.2 m*g* in older adults (Table 2). Children had higher BMIz compared with adolescents (0.62 ± 1.26 units vs 0.25 ± 1.08 units), which corresponded to the higher amount of overweight and obese participants in this age group (37% vs 24%). Although the mean BMI and waist circumference were almost equal in adults and older adults, the prevalence of overweight and obesity was greater by seven percent points in older adults (66%). The proportion of underweight participants was low (≤ 2%) across all age groups. Data on waist circumference were available for 71% of participants included in the final analytic sample.

**Table 2:** Characteristics of the study participants

	Children *n* = 2103		Adolescents *n* = 1234		Adults *n* = 1481		Older Adults *n* = 5000	
	Mean	SD	Mean	SD	Mean	SD	Mean	SD
Age (years)	9.4	1.3	16.1	1.3	55.5	13.6	72.0	4.5
Sex (*n*, % of *n*)								
Male	1027	48.8	592	48.0	892	60.2	3107	62.1
Female	1076	51.2	642	52.0	589	39.8	1893	37.9
Adiposity indicators								
BMI (kg/m^2^)	18.1	3.3	21.9	3.8	26.8	5.0	27.0	4.3
BMI *z*-score	0.62	1.26	0.25	1.08	N/A	N/A	N/A	N/A
Waist circumference (cm)^a^	61.5	8.8	N/A	N/A	96.1	12.5	96.0	11.8
Movement behaviors (min/day)^b^								
Sleep	545.2	35.3	439.7	38.8	474.1	40.4	468.2	40.1
Sedentary behavior	591.8	49.7	751.9	44.5	726.9	49.9	758.6	46.4
LPA	229.0	38.3	213.4	35.3	143.7	40.0	141.4	37.3
MVPA	74.0	76.7	35.0	81.4	95.3	69.7	71.8	76.2
Weight status (*n*, % of *n*)								
Underweight	24	1.1	14	1.1	23	1.6	50	1.0
Normal weight	1300	61.8	927	75.1	585	39.5	1657	33.1
Overweight	446	21.3	218	17.7	549	37.0	2280	45.6
Obese	333	15.8	75	6.1	324	21.9	1013	20.3
Socioeconomic status (*n*, % of *n*)								
Low	336	16.0	139	11.3	53	3.6	1659	33.2
Medium	556	26.4	581	47.1	731	49.4	2125	42.5
High	604	28.7	476	38.5	549	37.0	1214	24.3
Missing	607	28.9	38	3.1	148	10.0	2	< 0.1
Accelerometer characteristics								
Wear time (min/day)	1423.8	31.5	1413.2	36.2	1439.2	21.3	1440.4	16.3
Average acceleration (m*g*)	79.2	23.3	45.4	12.6	37.1	11.2	30.9	9.2
Number of valid days^c^	6	2	6	0	7	1	7	2

*BMI* body mass index, *LPA* light-intensity physical activity, *MVPA* moderate-to-vigorous physical activity, *N/A* not applicable, *SD* standard deviation

^a^There were missing data for this variable in children (*n* = 1216), adults (*n* = 179), and older adults (*n* = 7). No data were available for adolescents

^b^Values are presented as robust compositional mean and variance expressing the total variance related to a given time-use component

^c^Median and interquartile range

Estimated theoretical changes in BMIz and BMI associated with 30-min one-for-one reallocations between the components of the 24-h time-use composition are presented in Table 3. Increased obesity indicators were seen when participants substituted sleeping time for SB. Specifically, BMIz increased by 0.08 units (95% CI 0.04, 0.12) and 0.04 units (95% CI 0.001, 0.08) in children and adolescents, respectively, and BMI increased by 0.21 kg/m^2^ (95% CI 0.09, 0.33) in adults and 0.24 kg/m^2^ (95% CI 0.18, 0.30) in older adults, respectively. The BMIz also increased when time from sleep was relocated to LPA in children and adolescents. Except for adolescents, obesity indicators increased when participants substituted time spent in MVPA with any other movement behaviors. The greatest unfavorable changes were found when 30 min of MVPA was reallocated to LPA in children (0.26 units, 95% CI 0.15, 0.37) and to SB in adults (0.72 kg/m^2^, 95% CI 0.47, 0.96) and older adults (0.73 kg/m^2^, 95% CI 0.59, 0.87). In contrast, the greatest favorable changes in obesity indicators occurred when time spent in SB was replaced with time in MVPA in children (–0.15 units, 95% CI –0.21, –0.10), adults (–0.56 kg/m^2^, 95% CI –0.74, –0.39), and older adults (–0.52 kg/m^2^, 95% CI –0.61, –0.43). Several significant changes were also found for substituting LPA with other movement behaviors, but without any apparent pattern.

**Table 3:** Estimated theoretical changes in body mass index associated with 30-min reallocations of time between 24-h movement behaviors

	Difference (95% CI)^a^			
	↓ Sleep	↓ SB	↓ LPA	↓ MVPA
**Children (*n* = 2103)**				
↑ Sleep		** − 0.08 (− 0.11, − 0.04)**	** − 0.13 (− 0.18, − 0.07)**	**0.14 (0.05, 0.24)**
↑ SB	**0.08 (0.04, 0.12)**		** − 0.05 (− 0.10, − 0.01)**	**0.22 (0.13, 0.30)**
↑ LPA	**0.12 (0.07, 0.17)**	0.04 (− 0.002, 0.08)		**0.26 (0.15, 0.37)**
↑ MVPA	** − 0.08 (− 0.14, − 0.01)**	** − 0.15 (− 0.21, − 0.10)**	** − 0.20 (− 0.29, − 0.12)**	
**Adolescents (*n* = 1234)**				
↑ Sleep		− 0.04 (− 0.07, 0.001)	** − 0.09 (− 0.14, − 0.03)**	− 0.08 (− 0.35, 0.19)
↑ SB	**0.04 (0.001, 0.08)**		** − 0.05 (− 0.10, − 0.003)**	− 0.04 (− 0.30, 0.22)
↑ LPA	**0.08 (0.03, 0.13)**	**0.04 (0.001, 0.08)**		0.002 (− 0.29, 0.29)
↑ MVPA	0.05 (− 0.04, 0.14)	0.01 (− 0.07, 0.09)	− 0.04 (− 0.15, 0.07)	
**Adults (*n* = 1481)**				
↑ Sleep		** − 0.21 (− 0.33, − 0.09)**	0.08 (− 0.18, 0.34)	**0.51 (0.24, 0.78)**
↑ SB	**0.21 (0.09, 0.33)**		**0.29 (0.02, 0.55)**	**0.72 (0.47, 0.96)**
↑ LPA	− 0.05 (− 0.27, 0.18)	** − 0.26 (− 0.48, − 0.04)**		**0.46 (0.05, 0.86)**
↑ MVPA	** − 0.34 (− 0.56, − 0.13)**	** − 0.56 (− 0.74, − 0.39)**	− 0.27 (− 0.66, 0.11)	
**Older adults (*n* = 5000)**				
↑ Sleep		** − 0.24 (− 0.29, − 0.18)**	0.02 (− 0.09, 0.14)	**0.50 (0.35, 0.65)**
↑ SB	**0.24 (0.18, 0.30)**		**0.26 (0.14, 0.37)**	**0.73 (0.59, 0.87)**
↑ LPA	0.01 (− 0.09, 0.10)	** − 0.24 (− 0.33, − 0.14)**		**0.50 (0.29, 0.71)**
↑ MVPA	** − 0.28 (− 0.38, − 0.18)**	** − 0.52 (− 0.61, − 0.43)**	** − 0.26 (− 0.45, − 0.08)**	

Row variables represent behaviors that are being increased, while columns show behaviors being decreased. Cells show the effects and their CIs of replacing a column behavior by the corresponding row behavior

Bold values denote significant change in the obesity indicator

*CI* confidence interval, *LPA* light-intensity physical activity, *MVPA* moderate-to-vigorous physical activity, *SB* sedentary behavior, ↑ increased, **↓** decreased

^a^Change in body mass index *z*-score for children and adolescents and in body mass index (kg/m^2^) for adults and older adults

As shown in Tables S3 and S4 of the ESM, most changes remained statistically significant even with 10-min reallocations and became more clinically relevant when 60 min were reallocated. Furthermore, the gradient of changes was symmetric as an almost similar magnitude of changes was identified for opposite reallocations (Figs. S2–S5 of the ESM). For example, an almost identical magnitude of change in BMIz was found for 10-min (–0.03 vs + 0.03 units), 30-min (+ 0.08 vs –0.08 units), and 60-min reallocations (–0.15 vs + 0.16) between sleep and SB in children.

Table 4 presents theoretical changes in waist circumference associated with reallocation of 30 min of one movement behavior to another. Waist circumference increased when time spent in MVPA was reallocated to other movement behaviors in adults and older adults. The highest gain in waist circumference was observed when substituting MVPA with LPA in adults (2.66 cm, 95% CI 1.42, 3.90) and with SB in older adults (2.43 cm, 95% CI 2.07, 2.79). Substituting SB with MVPA in adults and with all other movement behaviors in older adults was associated with a significant decrease in waist circumference. There were also significant changes in waist circumference when time spent in sleep and LPA was reallocated in favor of other movement behaviors, but no apparent pattern was found. Changes in waist circumference remained significant even with 10-min reallocations and became more clinically relevant with 60-min reallocations as shown in Tables S5 and S6 of the ESM. The gradient of changes was asymmetric (Figs. S6–S8 of the ESM). For example, increasing MVPA at the expense of time spent in SB was associated with decreasing waist circumference by − 1.69 cm (95% CI –1.92, –1.46) in older adults, while gains in waist circumference by 2.43 cm (95% CI 2.07, 2.79) were found for an opposite reallocation.

**Table 4:** Estimated theoretical changes in waist circumference (cm) associated with 30-min reallocations of time between 24-h movement behaviors

	Difference (95% CI)			
	↓ Sleep	↓ SB	↓ LPA	↓ MVPA
**Children (*n* = 418)**^a^				
↑ Sleep		− 0.54 (− 1.18, 0.10)	** − 0.96 (− 1.87, − 0.06)**	0.36 (− 0.91, 1.64)
↑ SB	0.55 (− 0.10, 1.20)		− 0.43 (− 1.14, 0.28)	0.90 (− 0.16, 1.95)
↑ LPA	**0.91 (0.06, 1.76)**	0.35 (− 0.29, 0.99)		1.25 (− 0.19, 2.70)
↑ MVPA	− 0.10 (− 1.03, 0.83)	− 0.66 (− 1.35, 0.03)	− 1.08 (− 2.24, 0.08)	
**Adults (*n* = 1055)**				
↑ Sleep		− 0.35 (− 0.71, 0.01)	− 0.40 (− 1.20, 0.40)	**2.31 (1.49, 3.14)**
↑ SB	0.34 (− 0.02, 0.71)		− 0.07 (− 0.87, 0.74)	**2.65 (1.92, 3.39)**
↑ LPA	0.35 (− 0.33, 1.02)	− 0.01 (− 0.67, 0.66)		**2.66 (1.42, 3.90)**
↑ MVPA	** − 1.65 (− 2.28, − 1.02)**	** − 2.00 (− 2.53, − 1.47)**	** − 2.06 (− 3.24, − 0.88)**	
**Older adults (*n* = 4993)**				
↑ Sleep		** − 0.55 (− 0.69, − 0.40)**	− 0.15 (− 0.45, 0.15)	**1.90 (1.51, 2.29)**
↑ SB	**0.55 (0.40, 0.70)**		**0.38 (0.08, 0.69)**	**2.43 (2.07, 2.79)**
↑ LPA	0.18 (− 0.07, 0.43)	** − 0.38 (− 0.63, − 0.13)**		**2.06 (1.50, 2.62)**
↑ MVPA	** − 1.13 (− 1.40, –0.86)**	** − 1.69 (− 1.92, − 1.46)**	** − 1.30 (− 1.78, − 0.81)**	

Row variables represent behaviors that are being increased, while columns show behaviors being decreased. Cells show the effects and their CIs of replacing a column behavior by the corresponding row behavior

Bold values denote significant change in the obesity indicator

*CI* confidence interval, *LPA* light-intensity physical activity, *MVPA* moderate-to-vigorous physical activity, *SB* sedentary behavior, ↑ increased, ↓ decreased

^a^The analysis was performed on the dataset that included only participants with available socioeconomic status

## Discussion

In this meta-analysis of individual participant data, we estimated theoretical changes in obesity indicators associated with reallocations of time from one 24-h movement behavior to another. Our study has revealed that even modest reallocations of time (i.e., 10 min) are associated with significant changes in obesity indicators across the lifespan. Although most of the reallocation of time changes were consistent across age groups, some inconsistent results were identified for adolescents. The highest gain in obesity indicators was found when time allocated to MVPA was reallocated to other movement behaviors. In contrast, reallocations toward MVPA were associated with a decrease in obesity indicators. The highest magnitude of decrease in the obesity indicators was apparent when the time spent in SB was substituted with other movement behaviors. We found several significant changes for reallocations away from sleep and LPA, but there was no pattern apparent in all age groups. The magnitude of theoretical changes in BMI was nearly symmetric when opposite reallocations were compared, but asymmetric changes were found for waist circumference.

This study underscores the importance of optimizing daily movement behaviors to manage obesity effectively. In general, our findings are consistent with those of a recent scoping review analyzing associations between reallocations of time spent in 24-h movement behaviors and health outcomes [17]. Aligning with our findings, the review indicated that the most significant changes in health outcomes, including adiposity, occur with reallocations toward (i.e., positive changes) and away (i.e., negative changes) from MVPA. These findings strongly suggest that MVPA is a key component of 24-h movement behaviors and plays a critical role in the management of obesity across the lifespan. Interestingly, the lack of significant changes in adolescents highlights the complexity of obesity management in this specific stage of life. Potential reasons for this finding might be intensive hormonal changes and the influence of varying behavioral and environmental factors that are specifically associated with the development of adipose tissue during adolescence [39]. This emphasizes the need for strategies that account for critical life stages, ensuring that obesity prevention programs are effectively tailored for specific age groups.

By examining the role of reallocating time towards MVPA in the management of obesity, our study revealed an important finding about the variability in the dose–response relationship. For example, reallocating 10 min from MVPA to SB was associated with a 10% increase in BMIz in children, while a similar reallocation resulted in an increase in BMI of approximately 1% in adults and older adults. This discrepancy may be partly explained by the difference in physical activity patterns between age groups. Previous research [40] has shown that children are more likely to engage in physical activity of higher intensity than older individuals. The present study also identified differences in activity patterns by age. Average acceleration was highest among children and decreased for older participants. Additionally, intensity gradients were more negative in older participants (unpublished data), indicating that, with age, people sustain intense activity for shorter durations. These findings suggest that interventions targeting MVPA may need to be tailored to age-specific activity patterns to optimize obesity management across the lifespan.

Our study contributes to the growing body of evidence that emphasizes the importance of adequate sleep duration in obesity prevention [12, 13]. Favorable changes associated with increased sleep duration at the expense of SB observed in the present study highlight the potential benefits of maintaining good sleep habits. Therefore, practices such as avoiding behaviors that can delay bedtime and disrupt sleep consistency and quality (e.g., excessive screen time prior to sleep or consumption of energy drinks) should be considered when designing intervention strategies. The importance of sleep timing was emphasized in a study by Skjåkødegård and colleagues [41] who found that children with severe obesity tended to have a later mid-sleep time and more sleep problems compared with individuals with normal weight. The authors also highlighted that short sleep duration and later sleep timing is associated with obesogenic behaviors, characterized by increased screen time and reduced MVPA. Other studies have investigated this topic further, demonstrating that individuals with shorter sleep durations tend to allocate their additional waking hours to prolonged SB [42, 43]. This finding suggests a potential link between sleep deprivation and increased sedentary lifestyles, which could potentially contribute to obesity risk because of increased fatigue and reduced motivation to exercise. Future studies are warranted to investigate this topic more thoroughly as it was beyond the scope of the present study.

Evidence from cross-sectional and longitudinal studies [17, 44, 45] supports replacing sedentary time with physical activity as an effective approach to prevent obesity, especially among the adult population. The results of the present study align with these findings, showing the most significant reduction in obesity indicators when SB was replaced with other movement behaviors. Although the most beneficial changes in obesity indicators were observed for reallocations between SB and MVPA, reallocations from SB to low-intensity behaviors also showed favorable changes. Thus, our findings support a stepwise approach to increasing physical activity levels, beginning with reducing prolonged and uninterrupted SB by substituting with LPA [11]. Our results further suggest that even a modest reduction in SB can lead to meaningful improvements in both BMI and waist circumference. This highlights the potential benefits of interrupting prolonged SB with brief bouts of physical activity of any intensity, a behavioral change that could be particularly feasible in everyday life, including in various settings, and across diverse populations.

Beneficial associations of LPA with various health outcomes and mortality have been identified by a growing body of evidence [46]. Integrating low-intensity activities into daily routines can therefore contribute to obesity prevention, as it provides an opportunity to break up prolonged sedentary time and increase total physical activity. This behavioral change may be a first step toward an active lifestyle and may be feasible and sustainable even in people with severe obesity and its comorbidities [11]. However, the present study did not find a consistent trend in the changes in obesity indicators associated with reallocations of time spent in LPA. The favorable changes were found only when LPA increased at the expense of SB in adults and older adults. This result is in line with previous research showing that low-intensity daily activities contribute to obesity prevention in the adult population [47].

We found no favorable changes in obesity indicators when time was reallocated from other intensities to LPA in children and adolescents, suggesting that activities of higher intensity might be necessary to positively impact adiposity in the pediatric population. However, an increase in obesity indicators was associated with reallocating sleep time to LPA in these age groups. This finding questions the utility of incorporating LPA into strategies aimed at preventing childhood obesity. A meta-analysis by García-Hermoso and colleagues [45] partly answers this question by showing that increasing LPA at the expense of sedentary time did not lead to an improvement in adiposity outcomes. Given the varied findings in the present and previous studies, more research is needed to clearly determine how LPA can contribute to the management of obesity throughout the lifespan and identify the lowest intensity of activity that would result in beneficial changes in obesity indicators.

The present study demonstrates that optimizing all movement behaviors is required to achieve beneficial changes in obesity indicators, highlighting the need for a holistic approach to the management of obesity [14]. This is corroborated by existing evidence [15, 16], which indicates that adhering to 24-h movement recommendations is associated with a lower risk of obesity throughout life. A recent study [48] has explored this further and proposed a sweet-spot hypothesis [49] to determine the optimal daily durations of sleep, SB, LPA, and MVPA for maintaining healthy adiposity. Although no clear conclusion can be drawn from these studies, they outline a direction for future research and the development of public guidelines. These guidelines should consider the interactions between 24-h movement behaviors and their collective impact on health, with the aim of establishing a comprehensive approach to the management of obesity [14].

To facilitate the integration of our findings into public health messages, we estimated changes in obesity indicators using one-for-one reallocations as this approach is used most frequently in previous research [17]. Although such an approach allows simplification of complex results for straightforward messaging, it may not fully capture real-life behaviors. This could be particularly true for reallocating longer episodes of MVPA, as individuals are more likely to accumulate physical activity in short bursts rather than in long uninterrupted sessions [50, 51]. Therefore, employing a “one-for-remaining” reallocation approach (e.g., from sleep to SB, LPA, and MVPA) could more accurately represent real-life scenarios. This is because individuals generally distribute time from one behavior across multiple others, rather than to a single behavior [43]. However, future research is needed to investigate how individuals reallocate time in various real-life scenarios and to better understand how the use of time is influenced by multiple internal and external factors. Despite these limitations, we believe that our analysis supports the potential effectiveness of interventions focusing on behavioral change and offers a foundation for clear and understandable public health messages.

A main strength of this study is the use of a large and diverse sample of harmonized individual raw accelerometer data for a comprehensive pooled data meta-analysis, providing critical insights into the role of 24-h movement behaviors in the management of obesity across the lifespan. In addition, the original raw accelerometer data were auto-calibrated using local gravity as a reference, allowing accelerometer data from different geographic locations to be comparable.

The study’s limitations are primarily related to its initial design, which was not conceived as a multi-center study, potentially limiting the generalizability of the findings. Despite well-defined inclusion criteria, potential discrepancies in data collection methodologies, such as differences in the measurement of obesity indicators and differing accelerometer initialization rates, may introduce variability in the data, even though robust statistical adjustments have been employed to mitigate these effects. The use of age-specific absolute thresholds to determine the time spent in waking behaviors relies on the premise that these thresholds are universally applicable to all participants within each age group, regardless of sex, arm length, body composition, fitness level, or other individual characteristics. Therefore, it is reasonable to assume that some movement behaviors could potentially be misclassified as shown in previous studies [52, 53]. When interpreting our results, it is also important to consider that the present study does not allow direct comparisons of reallocation effects across age groups. This limitation arises from differences in the time spent in waking behaviors, which were derived using age- and device-specific absolute thresholds, as well as differences in the compositional means used as the starting point for reallocations. Finally, our analyses did not include a separate category for vigorous physical activity or specifically examine vigorous intermittent lifestyle physical activity, intensity categories that are associated with substantial health benefits and improved obesity indicators among various age groups [17, 54, 55]. Given the multi-dimensional nature of the 24-h movement behavior construct [56], future research should also incorporate additional dimensions such as posture- and domain-specific behaviors, in addition to intensity-specific behaviors.

## Conclusions

This comprehensive analysis of pooled individual participant data highlights the importance of reallocating time between 24-h movement behaviors in the management of obesity across the lifespan. The findings emphasize the crucial role of MVPA in reducing the risk of obesity and provide novel evidence of the importance of substituting SB with low-intensity movement behaviors, a lifestyle change that can be feasible and sustainable for a broad range of individuals, including those with severe obesity, low physical capabilities, and other associated health conditions. The most clinically relevant changes identified in this study were consistent across age groups, but some inconsistent findings were found for adolescents. These findings emphasize the need for lifestyle interventions tailored for specific age groups and illustrate the complexity of obesity management throughout life, thereby providing valuable guidance for intervention strategies targeting obesity.

## Supplementary Information

Below is the link to the electronic supplementary material.

Supplementary file1 (PDF 1426 KB)

Supplementary file2 (PDF 220 KB)

## Declarations

### Availability of Data and Material

The study protocol and analytic code are available on request from the corresponding author. Individual participant data used in this article were obtained, with permission, from original data custodians. These de-identified data may be available to researchers who provide a methodologically sound proposal. Requests for access to individual participant data can be e-mailed to Dr. Hartwig (timothy.hartwig@acu.edu.au). Data share agreements may need to be signed.

## References

[1] Koliaki C, Dalamaga M, Liatis S. Update on the obesity epidemic: after the sudden rise, is the upward trajectory beginning to flatten? Curr Obes Rep. 2023;12:514–27. 10.1007/s13679-023-00527-y. 37779155 10.1007/s13679-023-00527-y PMC10748771

[2] World Obesity Federation. World obesity atlas 2023. London: World Obesity Federation; 2023. Available from: https://data.worldobesity.org/publications/?cat=19. [Accessed 27 Nov 2024].

[3] Okunogbe A, Nugent R, Spencer G, Powis J, Ralston J, Wilding J. Economic impacts of overweight and obesity: current and future estimates for 161 countries. BMJ Glob Health. 2022;7: e009773. 10.1136/bmjgh-2022-009773. 36130777 10.1136/bmjgh-2022-009773 PMC9494015

[4] Swinburn BA, Sacks G, Hall KD, et al. The global obesity pandemic: shaped by global drivers and local environments. Lancet. 2011;378:804–14. 10.1016/S0140-6736(11)60813-1. 21872749 10.1016/S0140-6736(11)60813-1

[5] Loos RJF, Yeo GSH. The genetics of obesity: from discovery to biology. Nat Rev Genet. 2022;23:120–33. 10.1038/s41576-021-00414-z. 34556834 10.1038/s41576-021-00414-z PMC8459824

[6] Bray GA, Frühbeck G, Ryan DH, Wilding JPH. Management of obesity. Lancet. 2016;387:1947–56. 10.1016/S0140-6736(16)00271-3. 26868660 10.1016/S0140-6736(16)00271-3

[7] Wu T, Gao X, Chen M, Van Dam RM. Long-term effectiveness of diet-plus-exercise interventions vs. diet-only interventions for weight loss: a meta-analysis. Obes Rev. 2009;10:313–23. 10.1111/j.1467-789X.2008.00547.x. 19175510 10.1111/j.1467-789X.2008.00547.x

[8] Jensen SBK, Blond MB, Sandsdal RM, et al. Healthy weight loss maintenance with exercise, GLP-1 receptor agonist, or both combined followed by one year without treatment: a post-treatment analysis of a randomised placebo-controlled trial. eClinicalMedicine. 2024;69: 102475. 10.1016/j.eclinm.2024.102475. 38544798 10.1016/j.eclinm.2024.102475 PMC10965408

[9] Brown T, Moore THM, Hooper L, et al. Interventions for preventing obesity in children. Cochrane Database Syst Rev. 2019. 10.1002/14651858.CD001871.pub4. 31332776 10.1002/14651858.CD001871.pub4 PMC6646867

[10] Spiteri K, Broom D, Bekhet AH, de Caro JX, Laventure B, Grafton K. Barriers and motivators of physical activity participation in middle-aged and older-adults: a systematic review. J Aging Phys Act. 2019;27:929–44. 10.1123/japa.2018-0343. 31141447 10.1123/japa.2018-0343

[11] Dogra S, Copeland JL, Altenburg TM, Heyland DK, Owen N, Dunstan DW. Start with reducing sedentary behavior: a stepwise approach to physical activity counseling in clinical practice. Patient Educ Couns. 2022;105:1353–61. 10.1016/j.pec.2021.09.019. 34556383 10.1016/j.pec.2021.09.019

[12] Cappuccio FP, Taggart FM, Kandala N-B, et al. Meta-analysis of short sleep duration and obesity in children and adults. Sleep. 2008;31:619–26. 10.1093/sleep/31.5.619. 18517032 10.1093/sleep/31.5.619 PMC2398753

[13] Morrissey B, Taveras E, Allender S, Strugnell C. Sleep and obesity among children: a systematic review of multiple sleep dimensions. Pediatr Obes. 2020;15: e12619. 10.1111/ijpo.12619. 32072752 10.1111/ijpo.12619 PMC7154640

[14] Chaput JP, Saunders TJ, Carson V. Interactions between sleep, movement and other non-movement behaviours in the pathogenesis of childhood obesity. Obes Rev. 2017;18:7–14. 10.1111/obr.12508. 28164448 10.1111/obr.12508

[15] Rollo S, Antsygina O, Tremblay MS. The whole day matters: understanding 24-hour movement-guideline adherence and relationships with health indicators across the lifespan. J Sport Health Sci. 2020;9:493–510. 10.1016/j.jshs.2020.07.004. 32711156 10.1016/j.jshs.2020.07.004 PMC7749249

[16] López-Gil JF, Tapia-Serrano MA, Sevil-Serrano J, Sánchez-Miguel PA, García-Hermoso A. Are 24-hour movement recommendations associated with obesity-related indicators in the young population? A meta-analysis Obesity. 2023;31:2727–39. 10.1002/oby.23848. 37726964 10.1002/oby.23848

[17] Miatke A, Olds T, Maher C, et al. The association between reallocations of time and health using compositional data analysis: a systematic scoping review with an interactive data exploration interface. Int J Behav Nutr Phys Act. 2023;20:127. 10.1186/s12966-023-01526-x. 37858243 10.1186/s12966-023-01526-x PMC10588100

[18] Johnson DB, Gerstein DE, Evans AE, Woodward-Lopez G. Preventing obesity: a life cycle perspective. J Am Diet Assoc. 2006;106:97–102. 10.1016/j.jada.2005.09.048. 16390672 10.1016/j.jada.2005.09.048

[19] Dumuid D, Pedišić Ž, Stanford TE, et al. The compositional isotemporal substitution model: a method for estimating changes in a health outcome for reallocation of time between sleep, physical activity and sedentary behaviour. Stat Methods Med Res. 2018;28:846–57. 10.1177/0962280217737805. 10.1177/0962280217737805 29157152

[20] Hartwig T, Sanders T, Parker P, et al. The relationship between sleep and physical activity across the lifespan. 2022. Available from: https://www.osf.io/gzj9w. (Accessed 27 Nov 2024).

[21] Stewart LA, Clarke M, Rovers M, et al. Preferred reporting items for a systematic review and meta-analysis of individual participant data: the PRISMA-IPD statement. JAMA. 2015;313:1657–65. 10.1001/jama.2015.3656. 25919529 10.1001/jama.2015.3656

[22] Sanders T. SleepIPD. 2022. Available from: https://motivation-and-behaviour.github.io/sleepIPD. (Accessed 27 Nov 2024).

[23] Migueles JH, Rowlands AV, Huber F, Sabia S, van Hees VT. GGIR: a research community-driven open source R Package for generating physical activity and sleep outcomes from multi-day raw accelerometer data. J Meas Phys Behav. 2019;2:188. 10.1123/jmpb.2018-0063.

[24] van Hees VT, Sabia S, Jones SE, et al. Estimating sleep parameters using an accelerometer without sleep diary. Sci Rep. 2018;8:12975. 10.1038/s41598-018-31266-z. 30154500 10.1038/s41598-018-31266-z PMC6113241

[25] Hurter L, Fairclough SJ, Knowles ZR, Porcellato LA, Cooper-Ryan AM, Boddy LM. Establishing raw acceleration thresholds to classify sedentary and stationary behaviour in children. Children. 2018;5:172. 10.3390/children5120172. 30572683 10.3390/children5120172 PMC6306859

[26] Hildebrand M, Hansen BH, van Hees VT, Ekelund U. Evaluation of raw acceleration sedentary thresholds in children and adults. Scand J Med Sci Sports. 2017;27:1814–23. 10.1111/sms.12795. 27878845 10.1111/sms.12795

[27] Hildebrand M, van Hees VT, Hansen BH, Ekelund U. Age group comparability of raw accelerometer output from wrist- and hip-worn monitors. Med Sci Sports Exerc. 2014;46:1816–24. 10.1249/mss.0000000000000289. 24887173 10.1249/MSS.0000000000000289

[28] García-Hermoso A, Hormazábal-Aguayo I, Fernández-Vergara O, et al. A before-school physical activity intervention to improve cognitive parameters in children: the Active-Start study. Scand J Med Sci Sports. 2020;30:108–16. 10.1111/sms.13537. 31410887 10.1111/sms.13537

[29] Edney S, Plotnikoff R, Vandelanotte C, et al. “Active Team” a social and gamified app-based physical activity intervention: randomised controlled trial study protocol. BMC Public Health. 2017;17:859. 10.1186/s12889-017-4882-7. 29096614 10.1186/s12889-017-4882-7 PMC5667488

[30] Lubans DR, Smith JJ, Eather N, et al. Time-efficient intervention to improve older adolescents’ cardiorespiratory fitness: findings from the ‘Burn 2 Learn’ cluster randomised controlled trial. Br J Sports Med. 2020;55:751–8. 10.1136/bjsports-2020-103277. 33355155 10.1136/bjsports-2020-103277 PMC8223670

[31] Lonsdale C, Sanders T, Cohen KE, et al. Scaling-up an efficacious school-based physical activity intervention: study protocol for the ‘Internet-based Professional Learning to help teachers support Activity in Youth’ (iPLAY) cluster randomized controlled trial and scale-up implementation evaluation. BMC Public Health. 2016;16:873. 10.1186/s12889-016-3243-2. 27557641 10.1186/s12889-016-3243-2 PMC4997792

[32] Sánchez-López M, Ruiz-Hermosa A, Redondo-Tébar A, et al. Rationale and methods of the MOVI-da10! Study: a cluster-randomized controlled trial of the impact of classroom-based physical activity programs on children’s adiposity, cognition and motor competence. BMC Public Health. 2019;19:417. 10.1186/s12889-019-6742-0. 30999870 10.1186/s12889-019-6742-0 PMC6471851

[33] Harrex HAL, Skeaff SA, Black KE, et al. Sleep timing is associated with diet and physical activity levels in 9–11-year-old children from Dunedin, New Zealand: the PEDALS study. J Sleep Res. 2018;27: e12634. 10.1111/jsr.12634. 29160021 10.1111/jsr.12634

[34] Tercedor P, Villa-González E, Ávila-García M, et al. A school-based physical activity promotion intervention in children: rationale and study protocol for the PREVIENE Project. BMC Public Health. 2017;17:748. 10.1186/s12889-017-4788-4. 28950837 10.1186/s12889-017-4788-4 PMC5615806

[35] Cabanas-Sánchez V, Esteban-Cornejo I, Migueles JH, et al. Twenty four-hour activity cycle in older adults using wrist-worn accelerometers: the Seniors-ENRICA-2 study. Scand J Med Sci Sports. 2020;30:700–8. 10.1111/sms.13612. 31834945 10.1111/sms.13612

[36] da Costa BGG, Chaput J-P, Lopes MVV, Malheiros LEA, Tremblay MS, Silva KS. Prevalence and sociodemographic factors associated with meeting the 24-hour movement guidelines in a sample of Brazilian adolescents. PLoS One. 2020;15: e0239833. 10.1371/journal.pone.0239833. 32986765 10.1371/journal.pone.0239833 PMC7521749

[37] Rubín L, Gába A, Dygrýn J, Jakubec L, Materová E, Vencálek O. Prevalence and correlates of adherence to the combined movement guidelines among Czech children and adolescents. BMC Public Health. 2020;20:1692. 10.1186/s12889-020-09802-2. 33176735 10.1186/s12889-020-09802-2 PMC7661270

[38] Menai M, van Hees VT, Elbaz A, Kivimaki M, Singh-Manoux A, Sabia S. Accelerometer assessed moderate-to-vigorous physical activity and successful ageing: results from the Whitehall II study. Sci Rep. 2017;7:45772. 10.1038/srep45772. 10.1038/srep45772 PMC5377945 28367987

[39] Katzmarzyk PT, Shen W, Baxter-Jones A, et al. Adiposity in children and adolescents: correlates and clinical consequences of fat stored in specific body depots. Pediatr Obes. 2012;7:e42-61. 10.1111/j.2047-6310.2012.00073.x. 22911903 10.1111/j.2047-6310.2012.00073.x

[40] Steene-Johannessen J, Hansen BH, Dalene KE, et al. Variations in accelerometry measured physical activity and sedentary time across Europe: harmonized analyses of 47,497 children and adolescents. Int J Behav Nutr Phys Act. 2020;17:38. 10.1186/s12966-020-00930-x. 32183834 10.1186/s12966-020-00930-x PMC7079516

[41] Skjåkødegård HF, Danielsen YS, Frisk B, et al. Beyond sleep duration: sleep timing as a risk factor for childhood obesity. Pediatr Obes. 2021;16: e12698. 10.1111/ijpo.12698. 32729172 10.1111/ijpo.12698 PMC8809110

[42] Gába A, Dygrýn J, Štefelová N, et al. How do short sleepers use extra waking hours? A compositional analysis of 24-h time-use patterns among children and adolescents. Int J Behav Nutr Phys Act. 2020;17:104. 10.1186/s12966-020-01004-8. 32795287 10.1186/s12966-020-01004-8 PMC7427741

[43] Morrison S, Haszard JJ, Galland BC, et al. Where does the time go when children don’t sleep? A randomized crossover study. Obesity. 2023;31:625–34. 10.1002/oby.23615. 36575906 10.1002/oby.23615

[44] del Pozo-Cruz J, García-Hermoso A, Alfonso-Rosa RM, et al. Replacing sedentary time: meta-analysis of objective-assessment studies. Am J Prev Med. 2018;55:395–402. 10.1016/j.amepre.2018.04.042. 30122216 10.1016/j.amepre.2018.04.042

[45] García-Hermoso A, Saavedra JM, Ramírez-Vélez R, Ekelund U, del Pozo-Cruz B. Reallocating sedentary time to moderate-to-vigorous physical activity but not to light-intensity physical activity is effective to reduce adiposity among youths: a systematic review and meta-analysis. Obes Rev. 2017;18:1088–95. 10.1111/obr.12552. 28524399 10.1111/obr.12552

[46] Ross R, Janssen I, Tremblay MS. Public health importance of light intensity physical activity. J Sport Health Sci. 2024. 10.1016/j.jshs.2024.01.010. (**Epub ahead of print**). 38307207 10.1016/j.jshs.2024.01.010 PMC11282331

[47] Powell C, Browne LD, Carson BP, et al. Use of compositional data analysis to show estimated changes in cardiometabolic health by reallocating time to light-intensity physical activity in older adults. Sports Med. 2020;50:205–17. 10.1007/s40279-019-01153-2. 31350674 10.1007/s40279-019-01153-2

[48] Rasmussen CL, Gába A, Stanford T, et al. The Goldilocks Day for healthy adiposity measures among children and adolescents. Front Public Health. 2023;11:1–9. 10.3389/fpubh.2023.1158634. 10.3389/fpubh.2023.1158634 PMC10569221 37841713

[49] Holtermann A, Rasmussen CL, Hallman DM, Ding D, Dumuid D, Gupta N. 24-Hour physical behavior balance for better health for all: “the sweet-spot hypothesis.” Sports Med Open. 2021;7:98. 10.1186/s40798-021-00394-8. 34928441 10.1186/s40798-021-00394-8 PMC8688608

[50] Tarp J, Child A, White T, et al. Physical activity intensity, bout-duration, and cardiometabolic risk markers in children and adolescents. Int J Obes. 2018;42:1639–50. 10.1038/s41366-018-0152-8. 10.1038/s41366-018-0152-8 PMC6160399 30006582

[51] Jefferis BJ, Parsons TJ, Sartini C, et al. Does duration of physical activity bouts matter for adiposity and metabolic syndrome? A cross-sectional study of older British men. Int J Behav Nutr Phys Act. 2016;13:36. 10.1186/s12966-016-0361-2. 26980183 10.1186/s12966-016-0361-2 PMC4793648

[52] Trost SG, Brookes DSK, Ahmadi MN. Evaluation of wrist accelerometer cut-points for classifying physical activity intensity in youth. Front Digit Health. 2022;4: 884307. 10.3389/fdgth.2022.884307. 35585912 10.3389/fdgth.2022.884307 PMC9108175

[53] Weber KS, Godkin FE, Cornish BF, McIlroy WE, Van Ooteghem K. Wrist accelerometer estimates of physical activity intensity during walking in older adults and people living with complex health conditions: retrospective observational data analysis study. JMIR Form Res. 2023;7: e41685. 10.2196/41685. 36920452 10.2196/41685 PMC10131658

[54] Stamatakis E, Ahmadi MN, Gill JMR, et al. Association of wearable device-measured vigorous intermittent lifestyle physical activity with mortality. Nat Med. 2022;28:2521–9. 10.1038/s41591-022-02100-x. 36482104 10.1038/s41591-022-02100-x PMC9800274

[55] Grgic J, Dumuid D, Bengoechea EG, et al. Health outcomes associated with reallocations of time between sleep, sedentary behaviour, and physical activity: a systematic scoping review of isotemporal substitution studies. Int J Behav Nutr Phys Act. 2018;15:69. 10.1186/s12966-018-0691-3. 30001713 10.1186/s12966-018-0691-3 PMC6043964

[56] Stevens ML, Gupta N, Inan Eroglu E, et al. Thigh-worn accelerometry for measuring movement and posture across the 24-hour cycle: a scoping review and expert statement. BMJ Open Sport Exerc Med. 2020;6: e000874. 10.1136/bmjsem-2020-000874. 33408875 10.1136/bmjsem-2020-000874 PMC7768971

