# Supplementary material for: Reallocating Time Between 24-h Movement Behaviors for Obesity Management Across the Lifespan: A Pooled Data Meta-Analysis of More Than 9800 Participants from Seven Countries
Source: Sports Med. 2024 Dec 21;55(3):641–54. doi: 10.1007/s40279-024-02148-4 (PMC11985689; doi:10.1007/s40279-024-02148-4)
Supplement: Supplementary file 1 — Supplementary file1 (PDF 1426 KB) [file 40279_2024_2148_MOESM1_ESM.pdf]

Supplementary Files

Reallocating time between 24-hour movement behaviors for obesity management across the lifespan: A pooled data meta-analysis of more than 9,800 participants from seven countries

Contents

1 Flow of the study participants 3

Figure S1. Sankey diagram of the flow of the study participants 3

2 Identification and selection of confounding factors 4

3 Sensitivity analysis 4

Table S1. Sensitivity analysis comparing regression parameters for body mass index derived from the dataset without and with imputed missing values for socioeconomic status 5

Table S2. Sensitivity analysis comparing compositional regression parameters for waist circumference derived from the dataset without and with imputed missing values for socioeconomic status 6

4 Estimated changes in the obesity indicators associated with time reallocation between movement behaviors 7

Table S3. Estimated theoretical changes in body mass index associated with 10-minute reallocations of time between 24-h movement behaviors 7

Table S4. Estimated theoretical changes in body mass index associated with 60-minute reallocations of time between 24-h movement behaviors 8

Table S5. Estimated theoretical changes in waist circumference associated with 10-minute reallocations of time between 24-h movement behaviors 9

Table S6. Estimated theoretical changes in waist circumference associated with 60-minute reallocations of time between 24-h movement behaviors 10

Figure S2. Estimated difference in body mass index *z*-score associated with time reallocation between 24-hour movement behaviors among children 11

Figure S3. Estimated difference in body mass index *z*-score associated with time reallocation between 24-hour movement behaviors among adolescents 12

Figure S4. Estimated difference in body mass index associated with time reallocation between 24-hour movement behaviors among adults 13

Figure S5. Estimated difference in body mass index associated with time reallocation between 24-hour movement behaviors among older adults 14

Figure S6. Estimated difference in waist circumference associated with time reallocation between 24-hour movement behaviors among children 15

Figure S7. Estimated difference in waist circumference associated with time reallocation between 24-hour movement behaviors among adults 16

Figure S8. Estimated difference in waist circumference associated with time reallocation between 24-hour movement behaviors among older adults 17

5 Additional information 18
Figure A1. Violin plots of main variables of interest 18
Table A1. Multi-level multivariate regression model showing associations between compositional isometric log ratios and BMI across age groups (including covariates) 19
Table A2. Multi-level multivariate regression model showing associations between compositional isometric log ratios and waist circumference across age groups (including covariates) 20
6 References 21

1 Flow of the study participants

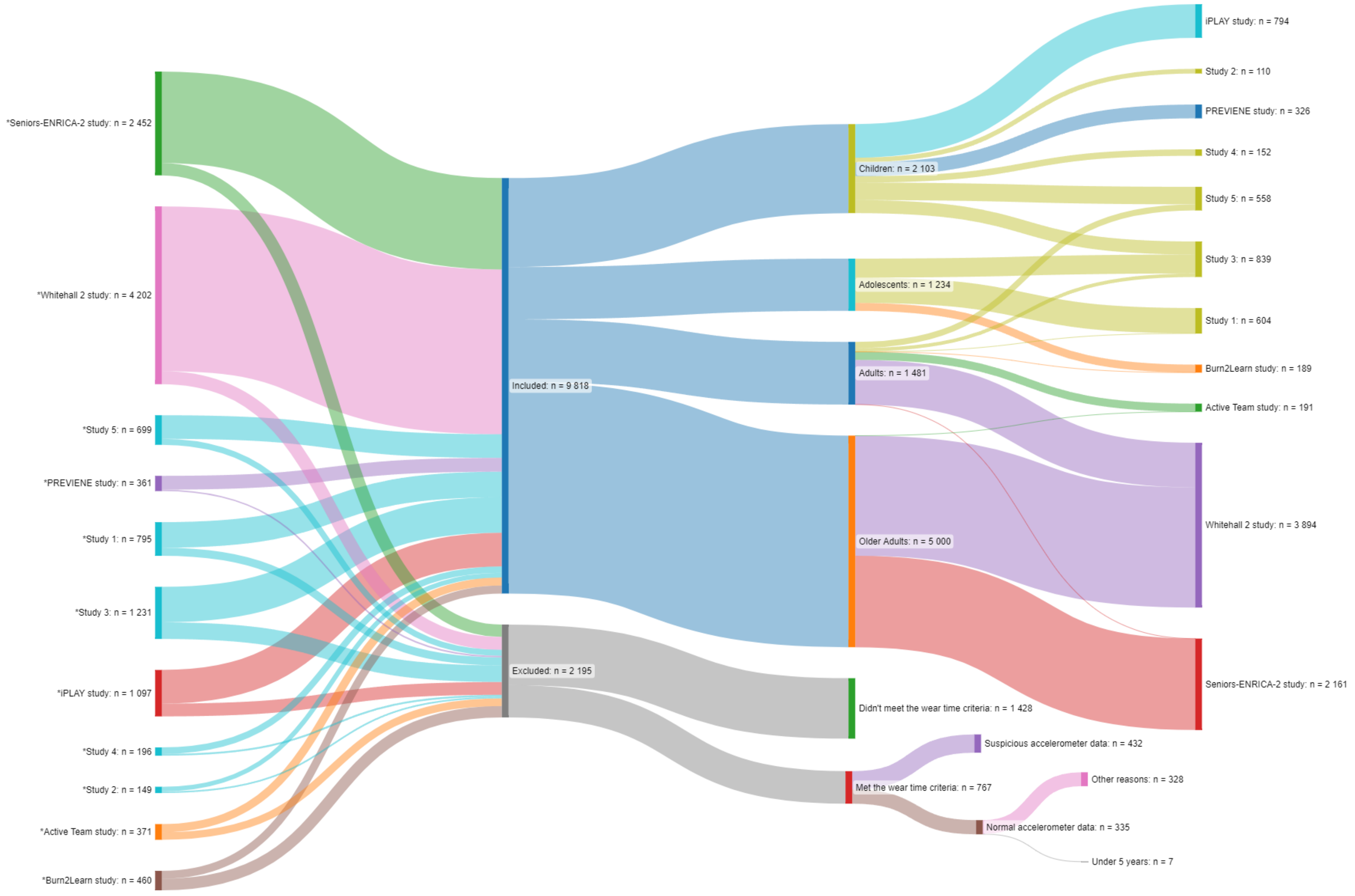

Figure S1. Sankey diagram of the flow of the study participants

2 Identification and selection of confounding factors

Potential confounders available in the harmonized dataset were selected to consider biological, demographic, and socioeconomical contributors to obesity. Age, sex, and socioeconomic status were included in all models based either on previous literature or significant association with obesity indicators (i.e., essential confounders). Because socioeconomic status was collected using various methods across studies, this variable was harmonized before analysis. Socioeconomic status was reported as a continuous variable with between 2 and 10 levels in all contributing studies. One study provided a government socioeconomic score (i.e., SEIFA score), while other studies used data on education status or the number of books at home as a proxy indicator of socioeconomic status. To make the studies comparable, the original variable was recoded as "Low", "Middle", and "High". Based on the data availability the following cut-points were used to categorize "Middle" socioeconomic status: 1) the SEIFA score ranged between 4 and 7 points, 2) the education status corresponded to completed secondary education or equivalent degree (mother's education status was used in participants under 19 years), or 3) having enough books at home to fill a bookcase (i.e., 26–100 books).

A total of 795 missing values for socioeconomic status occurred in the dataset. Although values for socioeconomic status were missing completely at random in most studies, they were completely missing by design in two studies. The iterative robust model-based imputation technique was selected to treat missing values [1]. The imputation algorithm bases the imputation on the regression where one of the chosen variables serves as a response with the remaining ones being the regressors. Therefore, several individual- and study-level predictors available in the dataset (e.g., BMI, physical activity volume and intensity, human development index) were selected to impute missing data. The imputation procedure was performed 100 times and 10 imputed datasets were generated in each iteration. A different response variable was chosen in each step, resulting in multiple imputed datasets which we then combined by Rubin's rules [2]. Based on the Akaike information criterion, socioeconomic status was included as a categorical covariate in models with BMIz/BMI as dependent variable and as numeric covariates in models with waist circumference.

Other individual-level (e.g., ethnicity, maturational status) and study-level data (e.g., Human development index, Gini index, Non-communicable disease policy implementation score [3]) were also considered as potential confounders. To include confounders in the final models, the following criteria had to be met: 1) the confounder must be harmonized across all included studies, 2) the confounder is significantly associated with obesity indicators, 3) the Akaike information criterion dropped after including the confounder in the model, and 4) the random intercept remains significant after the confounder is included in the model. Following these criteria, no additional confounders were included in the final models.

3 Sensitivity analysis

A sensitivity analysis was conducted to investigate a possible bias in the estimates due to the imputation of socioeconomic status. We performed a regression analysis on 1) the dataset in which participants without socioeconomic status were excluded and 2) the dataset in which missing values were imputed. This was done to ensure that the regression parameters were not affected by adding participants with imputed data. As shown in Table S1 and S2, there were no significant shifts in regression parameters for all models except for the model with waist circumference as a dependent variable in children. As guided by this finding, this regression model was the only model that was tested using the dataset from which participants with missing socioeconomic status were excluded.

Table S1. Sensitivity analysis comparing regression parameters for body mass index derived from the dataset without and with imputed missing values for socioeconomic status

	Children			Adolescents			Adults			Older Adults		
	β_{ilr1}	95% CI	*p*-value	β_{ilr1}	95% CI	*p*-value	β_{ilr1}	95% CI	*p*-value	β_{ilr1}	95% CI	*p*-value
**Dataset without missing values (*n* = 9023)**[a]												
Sleep (min/day)	–1.08	–1.69, –0.48	<0.001	–0.52	–0.95, –0.09	0.018	–0.75	–2.25, 0.76	0.330	–1.94	–2.57, –1.30	<0.001
SB (min/day)	0.71	0.24, 1.18	<0.001	0.09	–0.32, 0.49	0.674	3.15	1.68, 4.62	<0.001	3.84	3.18, 4.49	<0.001
LPA (min/day)	0.79	0.37, 1.21	<0.001	0.43	0.11, 0.75	0.009	–0.36	–1.60, 0.89	0.577	–0.62	–1.13, –0.10	0.019
MPVA (min/day)	–0.41	–0.65, –0.17	<0.001	0.004	–0.15, 0.16	0.958	–2.05	–2.85, –1.26	<0.001	–1.28	–1.58, –0.98	<0.001
**Dataset with imputed missing values (*n* = 9818)**												
Sleep (min/day)	–0.97	–1.44, –0.50	<0.001	–0.56	–0.99, –0.13	0.010	–1.34	–2.78, 0.10	0.068	–1.95	–2.58, –1.31	<0.001
SB (min/day)	0.71	0.33, 1.08	<0.001	0.12	–0.27, 0.52	0.547	3.85	2.46, 5.25	<0.001	3.85	3.19, 4.50	<0.001
LPA (min/day)	0.68	0.34, 1.02	<0.001	0.41	0.09, 0.73	0.011	–0.74	–1.93, 0.45	0.222	–0.61	–1.13, –0.10	0.019
MPVA (min/day)	–0.42	–0.61, –0.22	<0.001	0.03	–0.13, 0.18	0.737	–1.77	–2.53, –1.02	<0.001	–1.29	–1.59, –0.99	<0.001

CI – confidence interval*, ilr*1 – isometric log-ratio (the first coordinate), LPA – light intensity physical activity, MVPA – moderate-to-vigorous physical activity, SB – sedentary behavior.

[a] Socioeconomic status was not available for 607 children, 38 adolescents, 148 adults, and 2 older adults.

Body mass index *z*-score was used as the dependent variable in children and adolescents.

The 24-hour movement behaviors composition (independent variables) was expressed as the set of the first pivot coordinates which represents the relative contribution of one behavior relative to remaining behaviors.

All models were random intercept mixed models in which study identification number was entered as random effects. Models were adjusted for sex, age, and socioeconomic status for all age groups.

Table S2. Sensitivity analysis comparing compositional regression parameters for waist circumference derived from the dataset without and with imputed missing values for socioeconomic status

	Children			Adults			Older Adults		
	β_{ilr1}	95% CI	*p*-value	β_{ilr1}	95% CI	*p*-value	β_{ilr1}	95% CI	*p*-value
**Dataset without missing values (*n* = 6464)**[a]									
Sleep (min/day)	−7.60	−16.03, 0.84	0.078	−1.56	−5.79, 2.67	0.470	−4.24	−5.90, −2.57	<0.001
SB (min/day)	4.16	−2.13, 10.46	0.194	7.29	3.15, 11.44	0.001	9.14	7.41, 10.86	<0.001
LPA (min/day)	5.03	−0.32, 10.37	0.065	1.58	−2.06, 5.23	0.394	−0.37	−1.72, 0.99	0.596
MPVA (min/day)	−1.59	−4.08, 0.89	0.208	−7.32	−9.62, −5.02	<0.001	−4.53	−5.33, −3.74	<0.001
**Dataset with imputed missing values (*n* = 6935)**									
Sleep (min/day)	−6.91	−12.02, −1.80	0.008	−1.56	−5.79, 2.67	0.470	−4.23	−5.89, −2.57	<0.001
SB (min/day)	5.47	1.43, 9.52	0.008	7.29	3.15, 11.44	0.001	9.13	7.41, 10.85	<0.001
LPA (min/day)	3.04	−0.42, 6.49	0.085	1.58	−2.06, 5.23	0.394	−0.36	−1.72, 0.99	0.597
MPVA (min/day)	−1.60	−3.43, 0.24	0.088	−7.32	−9.62, −5.02	<0.001	−4.53	−5.33, −3.74	<0.001

CI – confidence interval, *ilr*1 – isometric log-ratio (the first coordinate), LPA – light-intensity physical activity, MVPA – moderate-to-vigorous physical activity, N/A – not applicable, SB – sedentary behavior.

[a] Socioeconomic status was not available for 469 children and 2 older adults. There were no missing data for adults.

The 24-hour movement behaviors composition (independent variables) was expressed as the set of the first pivot coordinates which represents the relative contribution of one behavior relative to remaining behaviors.

All models were random intercept mixed models in which study identification number was entered as random effects. Models were adjusted for sex, age, and socioeconomic status for all age groups.

4 Estimated changes in the obesity indicators associated with time reallocation between movement behaviors

Table S3. Estimated theoretical changes in body mass index associated with 10-minute reallocations of time between 24-h movement behaviors

	Change (95% CI)[a]							
	↓ Sleep		↓ SB		↓ LPA		↓ MVPA	
**Children (*n* = 2103)**								
↑ Sleep			**−0.03**	**(−0.04, −0.01)**	**−0.04**	**(−0.06, −0.02)**	**0.04**	**(0.01, 0.06)**
↑ SB	**0.03**	**(0.01, 0.04)**			**−0.02**	**(−0.03, −0.001)**	**0.06**	**(0.04, 0.09)**
↑ LPA	**0.04**	**(0.02, 0.06)**	**0.01**	**(0.001, 0.03)**			**0.08**	**(0.04, 0.11)**
↑ MVPA	**−0.03**	**(−0.05, −0.01)**	**−0.06**	**(−0.08, −0.04)**	**−0.07**	**(−0.10, −0.04)**		
**Adolescents (*n* = 1234)**								
↑ Sleep			**−0.01**	**(−0.02, −0.001)**	**−0.03**	**(−0.05, −0.01)**	−0.02	(−0.07, 0.03)
↑ SB	**0.01**	**(0.001, 0.02)**			**−0.02**	**(−0.03, −0.001)**	−0.01	(−0.05, 0.04)
↑ LPA	**0.03**	**(0.01, 0.04)**	**0.01**	**(0.001, 0.03)**			0.01	(−0.05, 0.06)
↑ MVPA	0.02	(−0.02, 0.05)	0.004	(−0.03, 0.04)	−0.01	(−0.05, 0.03)		
**Adults (*n* = 1481)**								
↑ Sleep			**−0.07**	**(−0.11, −0.03)**	0.02	(−0.06, 0.10)	**0.15**	**(0.06, 0.23)**
↑ SB	**0.07**	**(0.03, 0.11)**			**0.09**	**(0.01, 0.17)**	**0.22**	**(0.14, 0.29)**
↑ LPA	−0.02	(−0.10, 0.06)	**−0.09**	**(−0.17, −0.01)**			0.13	(−0.002, 0.26)
↑ MVPA	**−0.13**	**(−0.20, −0.05)**	**−0.20**	**(−0.26, −0.14)**	−0.11	(−0.23, 0.02)		
**Older Adults (*n* = 5000)**								
↑ Sleep			**−0.08**	**(−0.10, −0.06)**	0.003	(−0.03, 0.04)	**0.13**	**(0.09, 0.17)**
↑ SB	**0.08**	**(0.06, 0.10)**			**0.08**	**(0.05, 0.12)**	**0.21**	**(0.17, 0.25)**
↑ LPA	0.001	(−0.03, 0.03)	**−0.08**	**(−0.11, −0.05)**			**0.13**	**(0.07, 0.20)**
↑ MVPA	**−0.11**	**(−0.15, −0.07)**	**−0.19**	**(−0.22, −0.16)**	**−0.11**	**(−0.17, −0.04)**		

LPA – light-intensity physical activity, MVPA – moderate-to-vigorous physical activity, SB – sedentary behavior.
Row variables represent behaviors that are being increased, while columns show behaviors being decreased. Cells show the effects and their confidence intervals of replacing a column behavior by the corresponding row behavior.
Bold values denote significant change in the obesity indicator.
[a] Change in body mass index *z*-score for children and adolescents and in body mass index (kg/m^2) for adults and older adults.

Table S4. Estimated theoretical changes in body mass index associated with 60-minute reallocations of time between 24-h movement behaviors

	Change (95% CI)[a]							
	↓ Sleep		↓ SB		↓ LPA		↓ MVPA	
**Children (*n* = 2103)**								
↑ Sleep			**–0.15**	**(–0.23, –0.08)**	**–0.27**	**(–0.38, –0.15)**	**0.51**	**(0.22, 0.81)**
↑ SB	**0.16**	**(0.08, 0.23)**			**–0.12**	**(–0.22, –0.02)**	**0.66**	**(0.39, 0.93)**
↑ LPA	**0.23**	**(0.14, 0.33)**	0.07	(–0.01, 0.15)			**0.74**	**(0.41, 1.06)**
↑ MVPA	–0.12	(–0.24, 0.004)	**–0.28**	**(–0.37, –0.18)**	**–0.39**	**(–0.56, –0.22)**		
**Adolescents (*n* = 1234)**								
↑ Sleep			–0.07	(–0.14, 0.001)	**–0.18**	**(–0.29, –0.07)**	N/A[b]	
↑ SB	**0.08**	**(0.002, 0.16)**			**–0.11**	**(–0.21, –0.01)**	N/A[b]	
↑ LPA	**0.16**	**(0.06, 0.26)**	0.08	(–0.001, 0.16)			N/A[b]	
↑ MVPA	0.09	(–0.06, 0.24)	0.01	(–0.12, 0.15)	–0.09	(–0.30, 0.11)		
**Adults (*n* = 1481)**								
↑ Sleep			**–0.43**	**(–0.67, –0.19)**	0.21	(–0.39, 0.80)	**1.39**	**(0.69, 2.08)**
↑ SB	**0.42**	**(0.17, 0.67)**			**0.61**	**(0.02, 1.21)**	**1.79**	**(1.15, 2.43)**
↑ LPA	–0.07	(–0.48, 0.35)	**–0.51**	**(–0.92, –0.10)**			**1.30**	**(0.37, 2.23)**
↑ MVPA	**–0.59**	**(–0.98, –0.20)**	**–1.04**	**(–1.35, –0.72)**	–0.40	(–1.21, 0.40)		
**Older Adults (*n* = 5000)**								
↑ Sleep			**–0.48**	**(–0.59, –0.37)**	0.09	(–0.17, 0.35)	**1.81**	**(1.33, 2.29)**
↑ SB	**0.48**	**(0.37, 0.60)**			**0.55**	**(0.28, 0.81)**	**2.26**	**(1.80, 2.72)**
↑ LPA	0.04	(–0.14, 0.22)	**–0.46**	**(–0.64, –0.28)**			**1.82**	**(1.22, 2.42)**
↑ MVPA	**–0.45**	**(–0.63, –0.26)**	**–0.95**	**(–1.10, –0.80)**	**–0.38**	**(–0.76, –0.004)**		

LPA – light-intensity physical activity, MVPA – moderate-to-vigorous physical activity, N/A – not available, SB – sedentary behavior.

Row variables represent behaviors that are being increased, while columns show behaviors being decreased. Cells show the effects and their confidence intervals of replacing a column behavior by the corresponding row behavior.

Bold values denote significant change in the obesity indicator.

[a] Change in body mass index *z*-score for children and adolescents and in body mass index (kg/m^2) for adults and older adults.

[b] The estimated changes were not presented because the reallocations resulted in negative compositional elements (not suitable for the given methodology) due to the small proportion of moderate-to-vigorous physical activity compared to the estimated changes.

Table S5. Estimated theoretical changes in waist circumference associated with 10-minute reallocations of time between 24-h movement behaviors

	Change (95% CI)							
	↓ Sleep		↓ SB		↓ LPA		↓ MVPA	
**Children (*n* = 418)**[a]								
↑ Sleep			–0.18	(–0.40, 0.03)	**–0.31**	**(–0.61, –0.02)**	0.08	(–0.29, 0.45)
↑ SB	0.18	(–0.03, 0.40)			–0.13	(–0.36, 0.09)	0.26	(–0.03, 0.56)
↑ LPA	**0.31**	**(0.02, 0.60)**	0.12	(–0.10, 0.35)			0.39	(–0.05, 0.82)
↑ MVPA	–0.05	(–0.39, 0.28)	–0.24	(–0.49, 0.02)	–0.37	(–0.77, 0.03)		
**Adults (*n* = 1055)**								
↑ Sleep			–0.12	(–0.24, 0.004)	–0.13	(–0.38, 0.12)	**0.67**	**(0.43, 0.92)**
↑ SB	0.12	(–0.01, 0.24)			–0.01	(–0.26, 0.24)	**0.79**	**(0.57, 1.00)**
↑ LPA	0.12	(–0.11, 0.36)	0.005	(–0.23, 0.24)			**0.80**	**(0.40, 1.19)**
↑ MVPA	**–0.60**	**(–0.83, –0.38)**	**–0.72**	**(–0.91, –0.53)**	**–0.73**	**(–1.12, –0.34)**		
**Older Adults (*n* = 4993)**								
↑ Sleep			**–0.18**	**(–0.23, –0.13)**	–0.05	(–0.15, 0.04)	**0.51**	**(0.40, 0.62)**
↑ SB	**0.18**	**(0.13, 0.23)**			**0.13**	**(0.03, 0.22)**	**0.69**	**(0.59, 0.79)**
↑ LPA	0.06	(–0.03, 0.15)	**–0.13**	**(–0.22, –0.04)**			**0.57**	**(0.40, 0.74)**
↑ MVPA	**–0.43**	**(–0.53, –0.34)**	**–0.62**	**(–0.70, –0.53)**	**–0.49**	**(–0.65, –0.32)**		

LPA – light-intensity physical activity, MVPA – moderate-to-vigorous physical activity, SB – sedentary behavior.
Row variables represent behaviors that are being increased, while columns show behaviors being decreased. Cells show the effects and their confidence intervals of replacing a column behavior by the corresponding row behavior.
Bold values denote significant change in the obesity indicator.
[a] The analysis was performed on the dataset which included only participants with available socioeconomic status.

Table S6. Estimated theoretical changes in waist circumference associated with 60-minute reallocations of time between 24-h movement behaviors

	Change (95% CI)							
	↓ Sleep		↓ SB		↓ LPA		↓ MVPA	
**Children (*n* = 418)**[a]								
↑ Sleep			−1.07	(−2.35, 0.21)	**−2.01**	**(−3.90, −0.12)**	1.61	(−2.23, 5.45)
↑ SB	1.12	(−0.20, 2.43)			−0.97	(−2.49, 0.54)	2.65	(−0.79, 6.08)
↑ LPA	**1.78**	**(0.12, 3.44)**	0.63	(−0.61, 1.86)			3.31	(−0.83, 7.44)
↑ MVPA	−0.05	(−1.76, 1.66)	−1.20	(−2.42, 0.01)	−2.14	(−4.40, 0.11)		
**Adults (*n* = 1055)**								
↑ Sleep			−0.70	(−1.41, 0.003)	−0.90	(−2.72, 0.91)	**6.14**	**(4.04, 8.23)**
↑ SB	0.68	(−0.06, 1.43)			−0.24	(−2.06, 1.58)	**6.80**	**(4.87, 8.73)**
↑ LPA	0.66	(−0.61, 1.93)	−0.07	(−1.31, 1.18)			**6.78**	**(3.93, 9.63)**
↑ MVPA	**−2.91**	**(−4.08, −1.75)**	**−3.64**	**(−4.59, −2.69)**	**−3.84**	**(−6.31, −1.36)**		
**Older Adults (*n* = 4993)**								
↑ Sleep			**−1.09**	**(−1.38, −0.81)**	−0.27	(−0.95, 0.42)	**6.65**	**(5.38, 7.91)**
↑ SB	**1.10**	**(0.80, 1.40)**			**0.78**	**(0.08, 1.47)**	**7.69**	**(6.47, 8.90)**
↑ LPA	0.39	(−0.08, 0.86)	**−0.76**	**(−1.24, −0.29)**			**6.98**	**(5.40, 8.55)**
↑ MVPA	**−1.88**	**(−2.36, −1.40)**	**−3.04**	**(−3.44, −2.63)**	−2.21	(−3.21, −1.21)		

LPA – light-intensity physical activity, MVPA – moderate-to-vigorous physical activity, SB – sedentary behavior.
Row variables represent behaviors that are being increased, while columns show behaviors being decreased. Cells show the effects and their confidence intervals of replacing a column behavior by the corresponding row behavior.
Bold values denote significant change in the obesity indicator.
[a] The analysis was performed on the dataset which included only participants with available socioeconomic status.

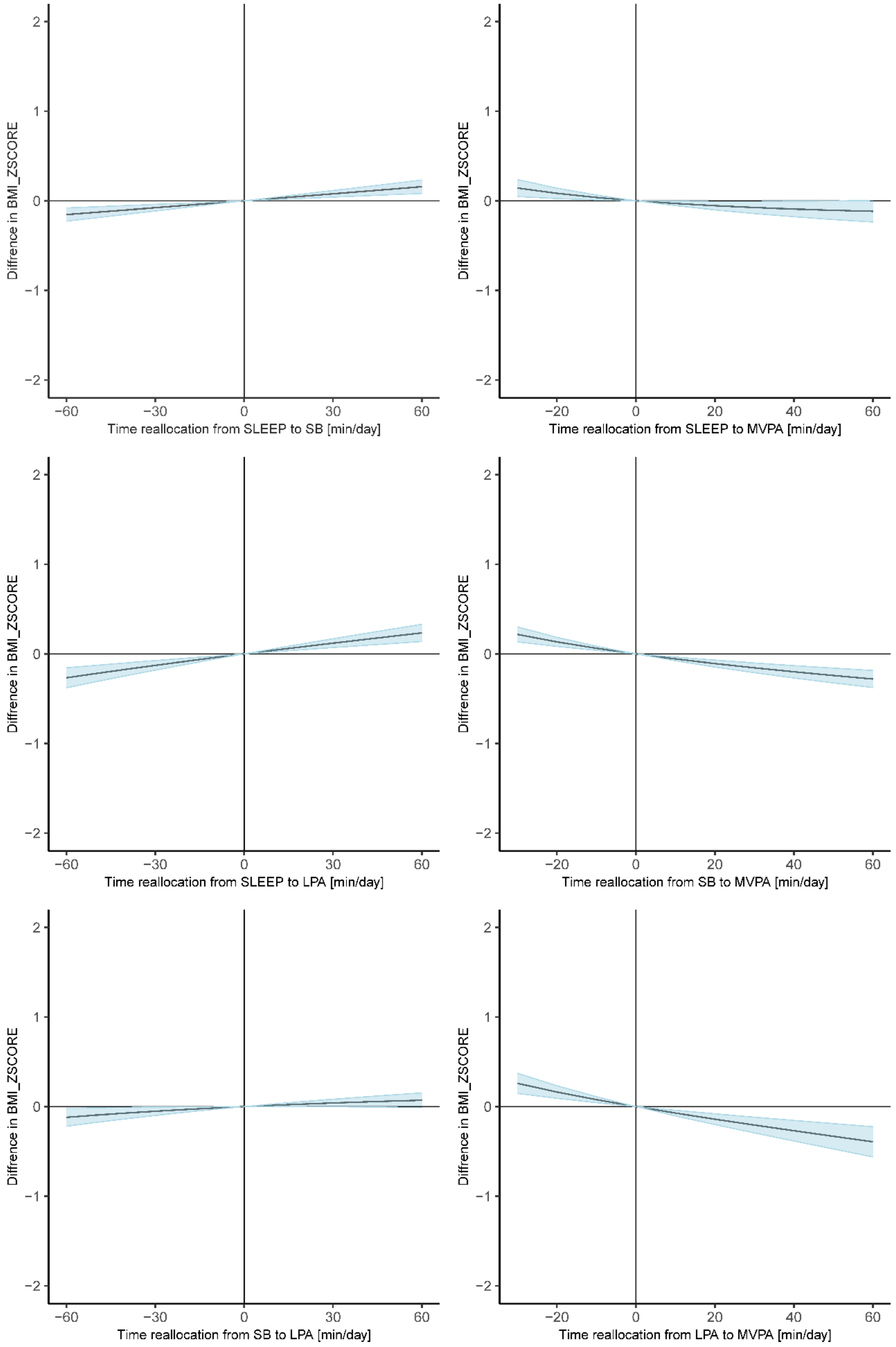

Figure S2. Estimated difference in body mass index *z*-score associated with time reallocation between 24-hour movement behaviors among children

BMI – body mass index, LPA – light-intensity physical activity, SB – sedentary behavior, MVPA – moderate-to-vigorous physical activity.

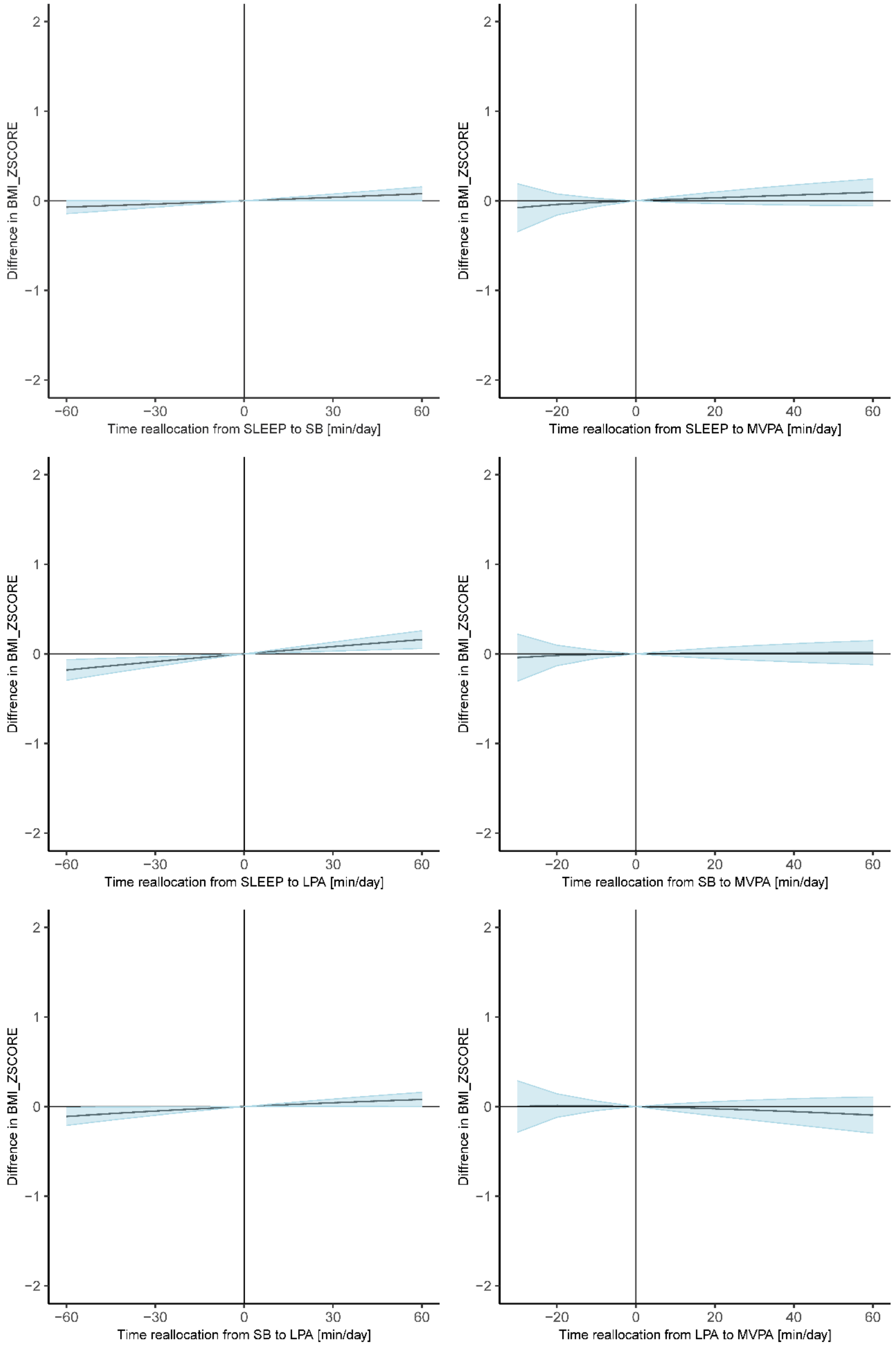

Figure S3. Estimated difference in body mass index *z*-score associated with time reallocation between 24-hour movement behaviors among adolescents

BMI – body mass index, LPA – light-intensity physical activity, SB – sedentary behavior, MVPA – moderate-to-vigorous physical activity.

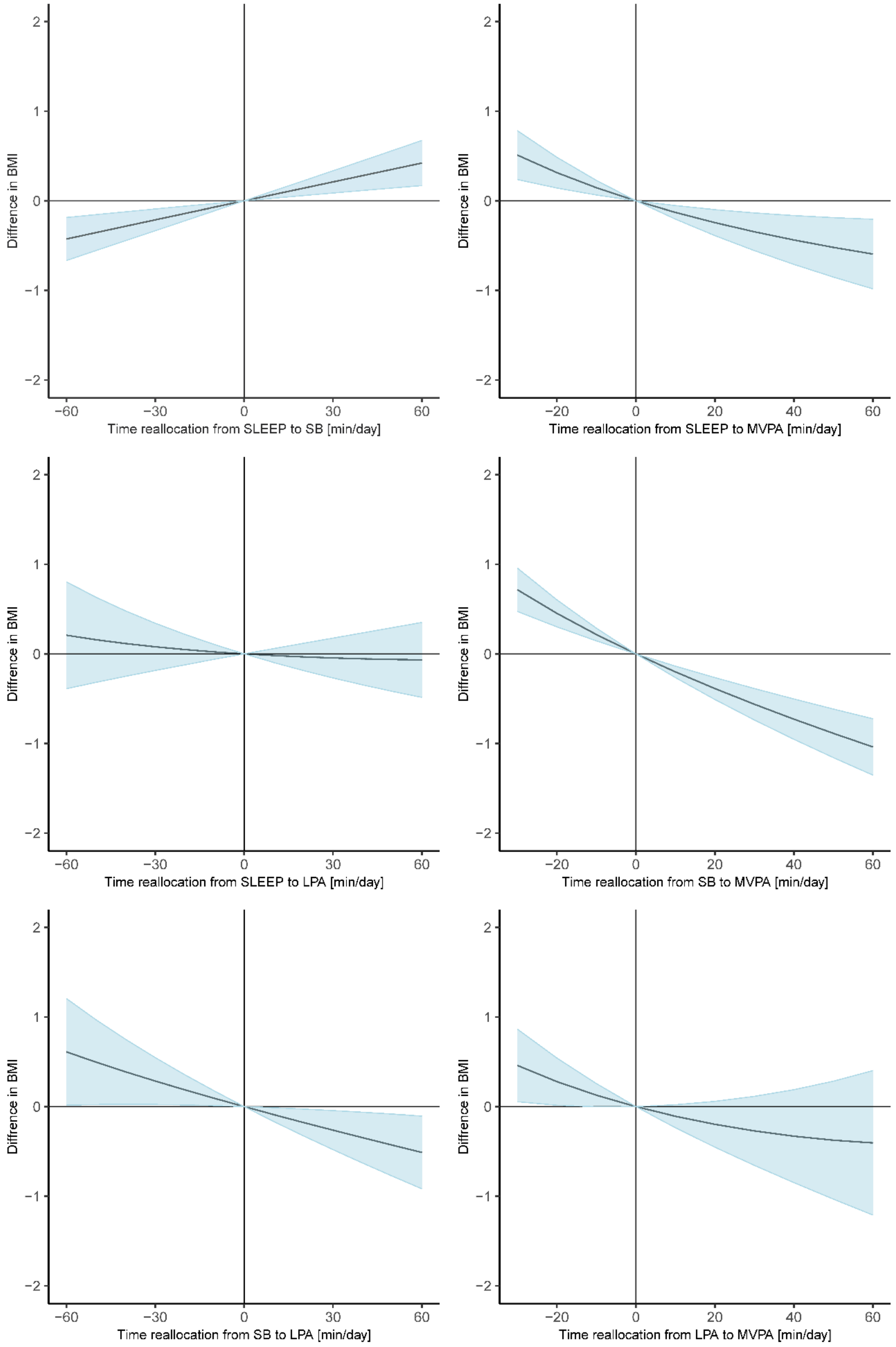

Figure S4. Estimated difference in body mass index associated with time reallocation between 24-hour movement behaviors among adults

BMI – body mass index, LPA – light-intensity physical activity, SB – sedentary behavior, MVPA – moderate-to-vigorous physical activity

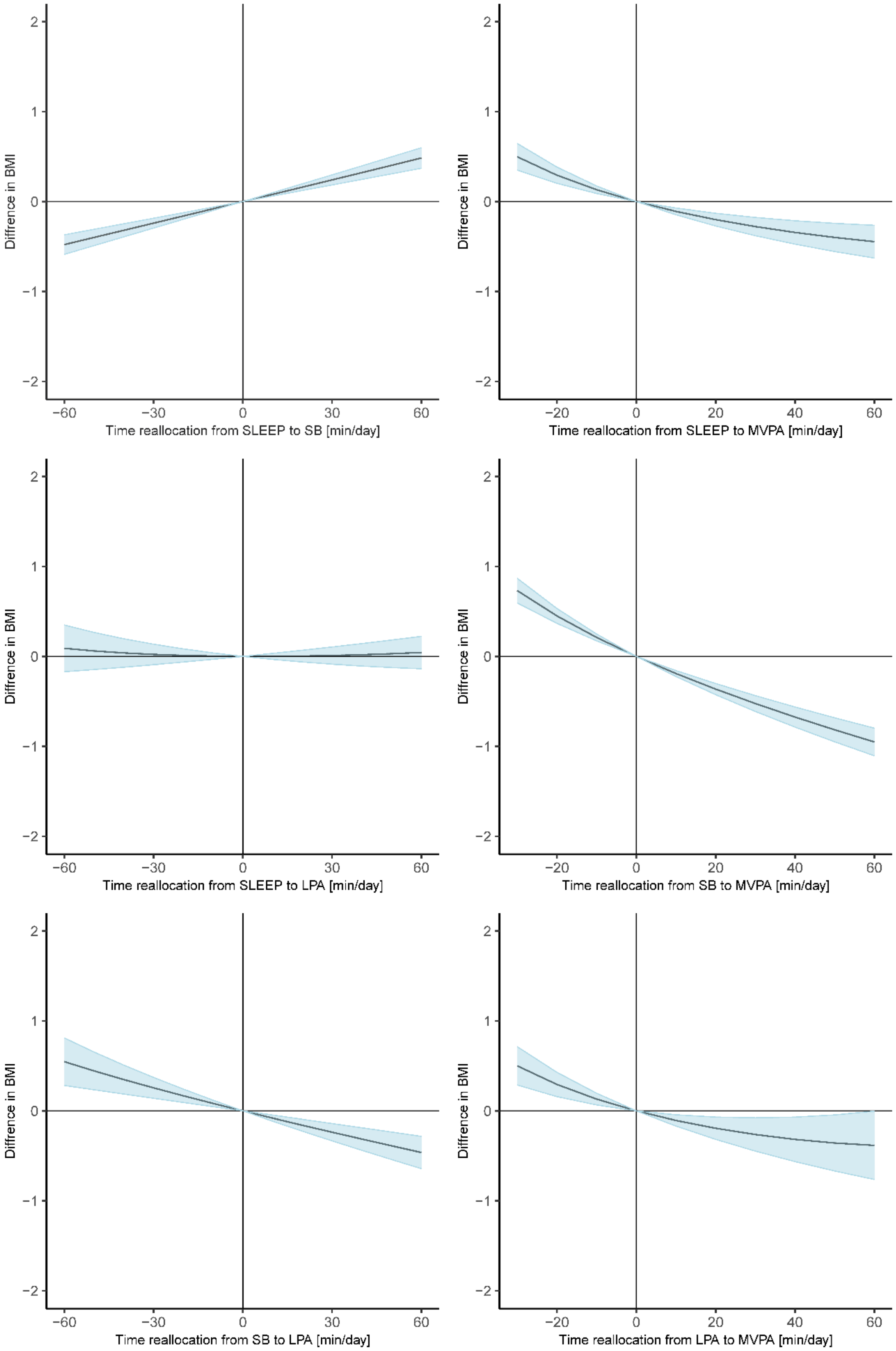

Figure S5. Estimated difference in body mass index associated with time reallocation between 24-hour movement behaviors among older adults

BMI – body mass index, LPA – light-intensity physical activity, SB – sedentary behavior, MVPA – moderate-to-vigorous physical activity

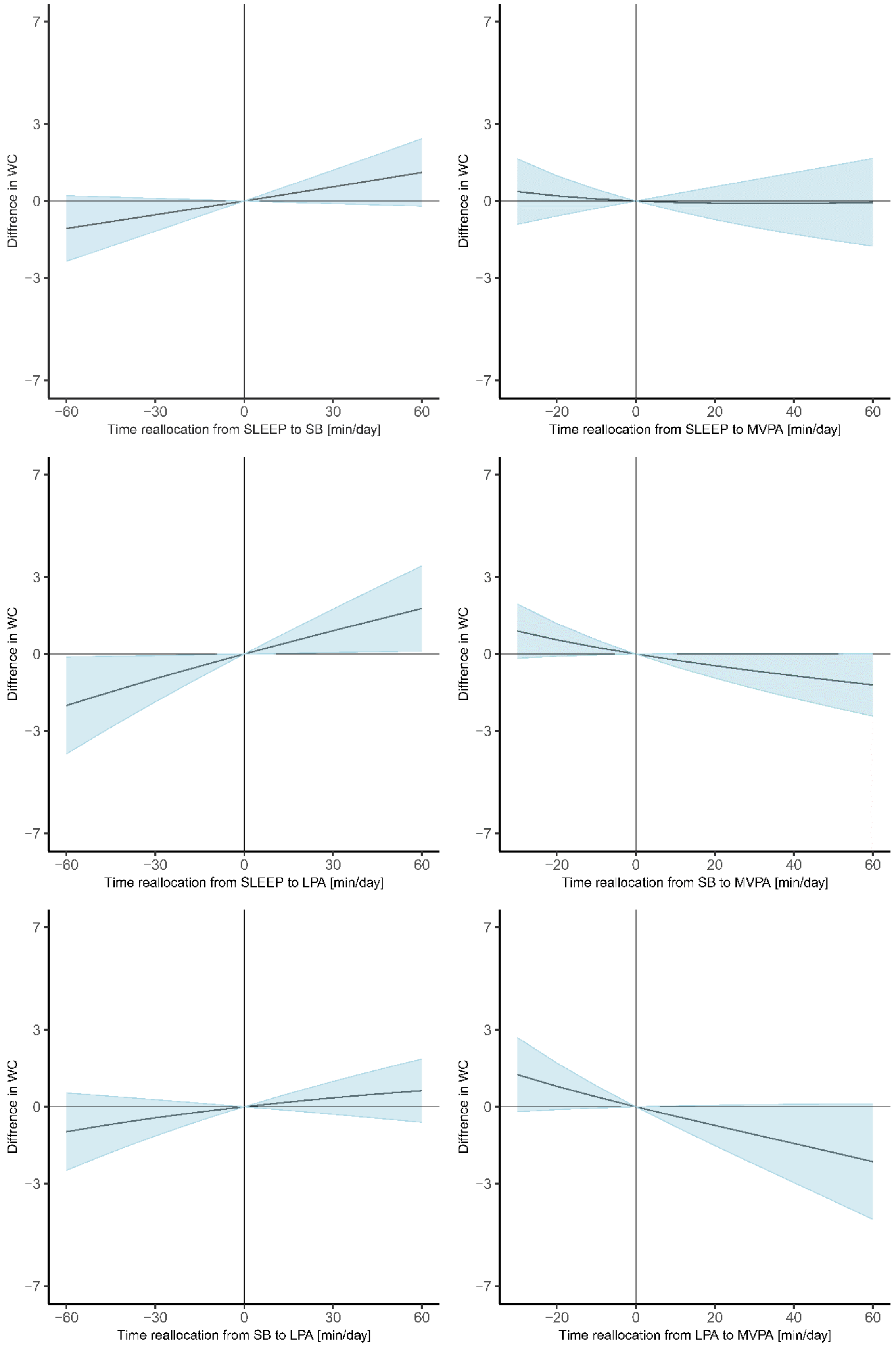

Figure S6. Estimated difference in waist circumference associated with time reallocation between 24-hour movement behaviors among children

LPA – light-intensity physical activity, SB – sedentary behavior, MVPA – moderate-to-vigorous physical activity, WC – waist circumference

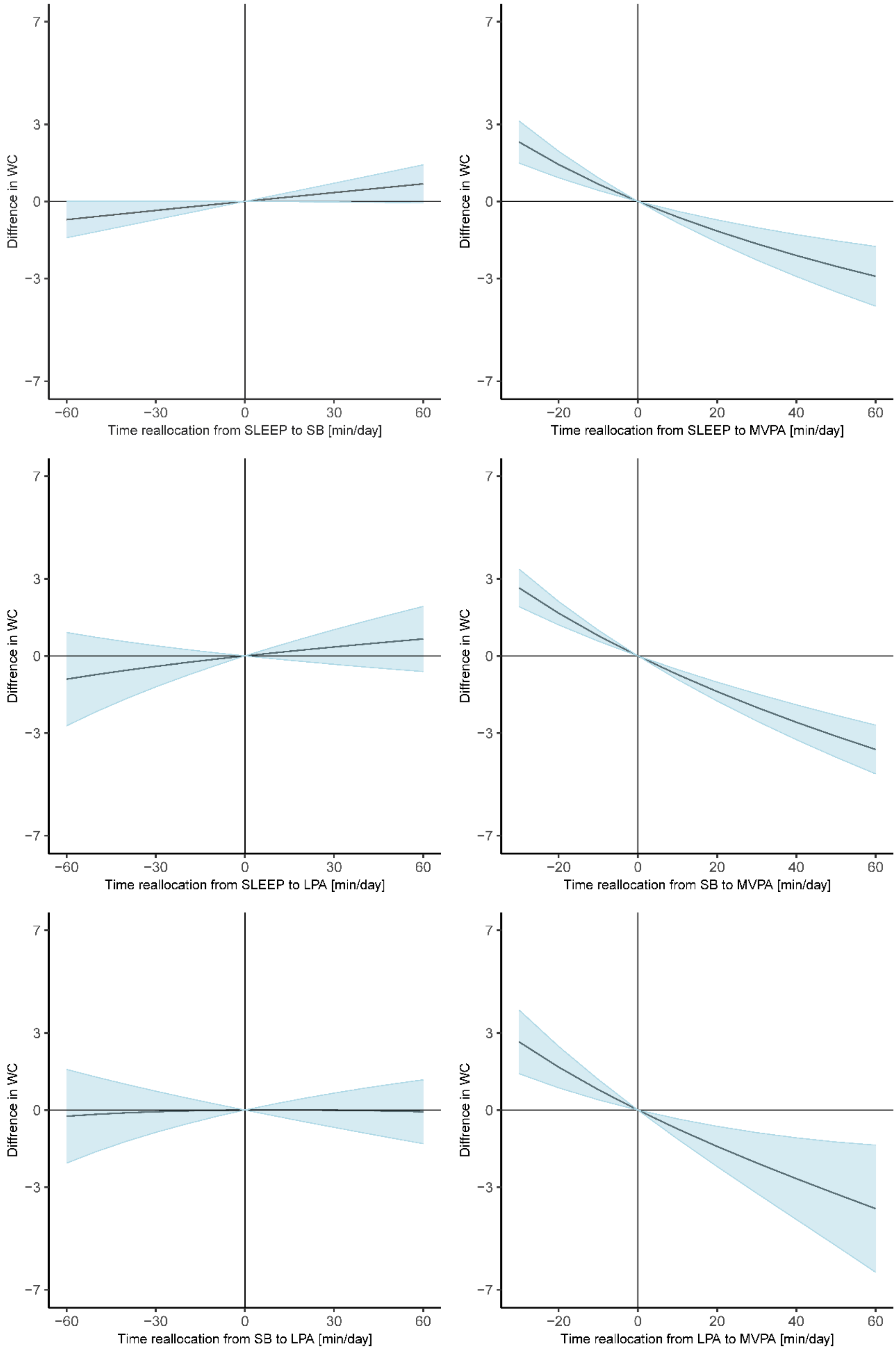

Figure S7. Estimated difference in waist circumference associated with time reallocation between 24-hour movement behaviors among adults

LPA – light-intensity physical activity, SB – sedentary behavior, MVPA – moderate-to-vigorous physical activity, WC – waist circumference

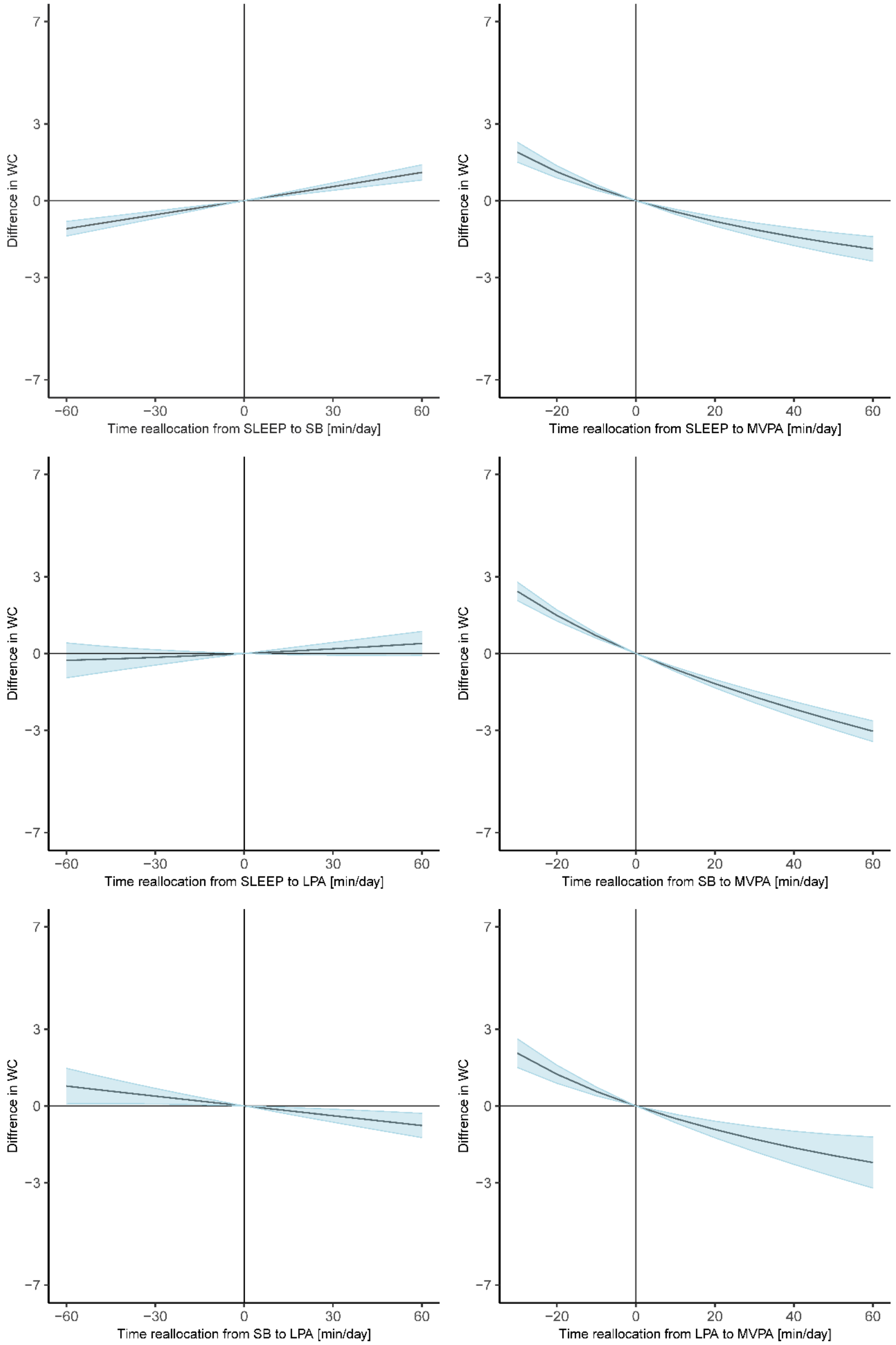

Figure S8. Estimated difference in waist circumference associated with time reallocation between 24-hour movement behaviors among older adults

LPA – light-intensity physical activity, SB – sedentary behavior, MVPA – moderate-to-vigorous physical activity, WC – waist circumference

5 Additional information

This section provides additional information to enhance transparency in the statistical methods [4] used in this study. The figure and tables aim to support the assessment of data assumptions and model fit, ensuring adherence to best practices in medical research reporting.

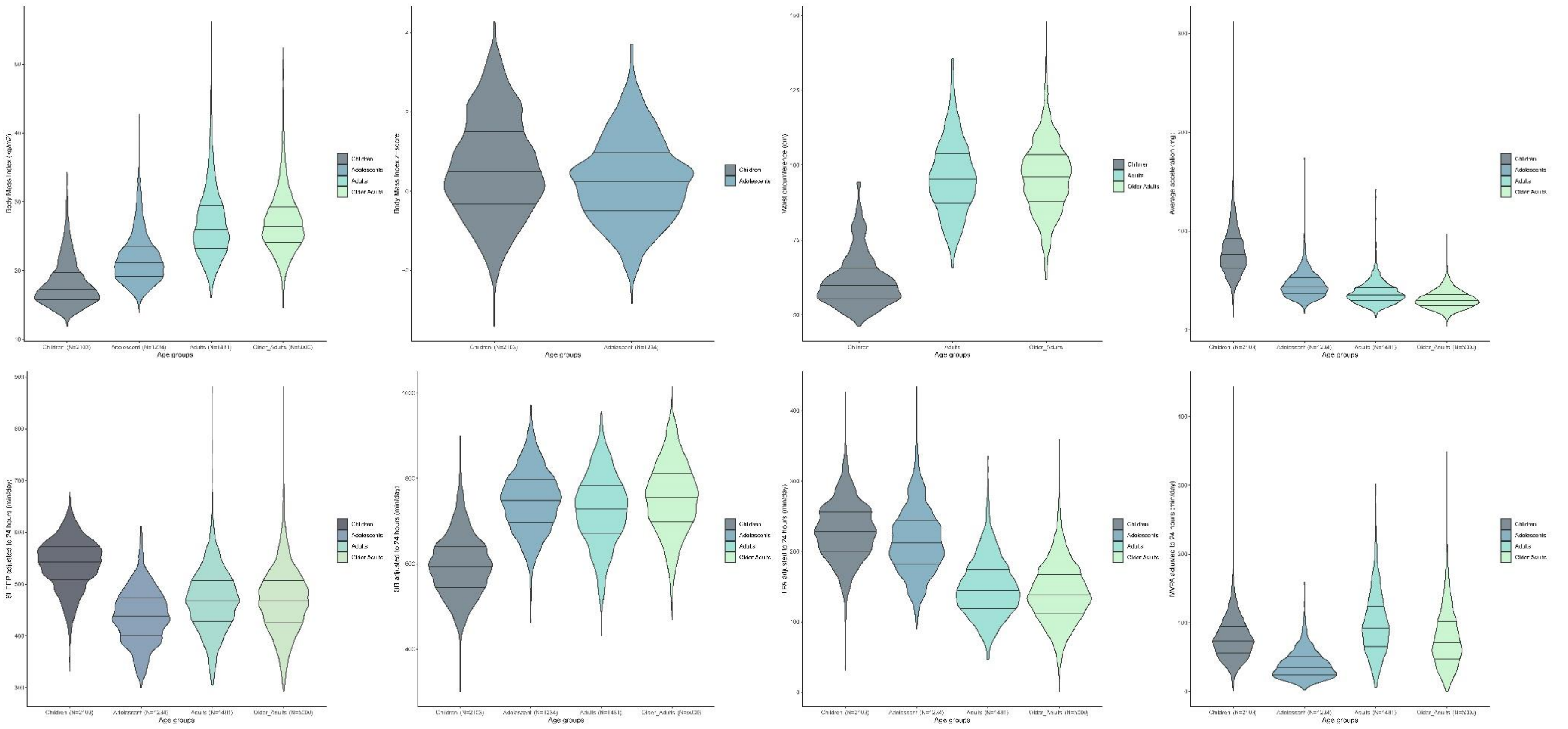

Figure A1. Violin plots of main variables of interest

LPA – light-intensity physical activity, SB – sedentary behavior, MVPA – moderate-to-vigorous physical activity, WC – waist circumference.

Table A1. Multi-level multivariate regression model showing associations between compositional isometric log ratios and BMI across age groups (including covariates)

	Children[a] Marginal R^2 = 0.03 Conditional R^2 = 0.15				Adolescents[a] Marginal R^2 = 0.02 Conditional R^2 = 0.06				Adults Marginal R^2 = 0.07 Conditional R^2 = 0.36				Older Adults Marginal R^2 = 0.08 Conditional R^2 = 0.09			
	β	95% CI lower	95% CI upper	*p*-value	β	95% CI lower	95% CI upper	*p*-value	β	95% CI lower	95% CI upper	*p*-value	β	95% CI lower	95% CI upper	*p*-value
Intercept	0.95	0.25	1.65	0.008	1.07	0.18	1.95	0.019	18.15	14.22	22.08	<0.001	31.14	29.09	33.19	<0.001
$Sleep_{ilr1}$	–0.97	–1.44	–0.50	<0.001	–0.56	–0.99	–0.13	0.010	–1.34	–2.78	0.10	0.068	–1.95	–2.58	–1.31	<0.001
SB_{ilr1}	0.71	0.33	1.08	<0.001	0.12	–0.27	0.52	0.547	3.85	2.46	5.25	<0.001	3.85	3.19	4.50	<0.001
LPA_{ilr1}	0.68	0.34	1.02	<0.001	0.41	0.09	0.73	0.011	–0.74	–1.93	0.45	0.222	–0.61	–1.13	–0.10	0.019
$MVPA_{ilr1}$	–0.42	–0.61	–0.22	<0.001	0.03	–0.13	0.18	0.737	–1.77	–2.53	–1.02	<0.001	–1.29	–1.59	–0.99	<0.001
SES[b]	–0.07	–0.15	0.01	0.097	–0.11	–0.21	–0.02	0.014	–0.55	–1.00	–0.09	0.018	–0.82	–0.98	–0.65	<0.001
Sex	–0.18	–0.30	–0.07	0.002	0.10	–0.02	0.23	0.097	0.11	–0.45	0.66	0.705	0.23	–0.03	0.49	0.089
Age	–0.04	–0.10	0.01	0.133	–0.02	–0.07	0.03	0.346	0.09	0.04	0.14	<0.001	–0.11	–0.14	–0.08	<0.001
Study ID (random)	0.46	0.24	0.88	<0.001	0.22	0.07	0.65	<0.001	3.13	1.36	7.16	<0.001	0.40	0.09	1.67	<0.001

CI – confidence interval, ID – identification number, *ilr*1 – isometric log-ratio (the first coordinate), LPA – light-intensity physical activity, MVPA – moderate-to-vigorous physical activity, SB – sedentary behavior, SES – socioeconomic status.
The final model aggregates individual models, each containing a complete set of pivot coordinates (i.e., *ilr*1, *ilr*2, and *ilr*3). Four individual models were built, rearranging the component order to express each behavior as *ilr*1. The final model combines the first pivot coordinates (*ilr*1) from these individual models.

Marginal R^2 represents the variance explained by the fixed effects, as calculated using the equation by Nakagawa and Schielzeth [5].

Conditional R^2 represents the variance explained by the entire model, including both fixed and random effects, as calculated using the equation by Nakagawa and Schielzeth [5].

[a] BMI *z*-score was used in children and adolescents.

[b] Socioeconomic status was included as a continuous variable in the analysis.

Table A2. Multi-level multivariate regression model showing associations between compositional isometric log ratios and waist circumference across age groups (including covariates)

	Children[a] Marginal R^2 = 0.12 Conditional R^2 = 0.20				Adults Marginal R^2 = 0.14 Conditional R^2 = 0.13				Older Adults Marginal R^2 = 0.17 Conditional R^2 = 0.17			
	β	95% CI lower	95% CI upper	*p*-value	β	95% CI lower	95% CI upper	*p*-value	β	95% CI lower	95% CI upper	*p*-value
Intercept	41.47	33.39	49.55	<0.001	62.15	27.34	96.96	<0.001	105.97	100.71	111.24	<0.001
$Sleep_{ilr1}$	–7.60	–16.03	0.84	0.078	–1.56	–5.79	2.67	0.470	–4.23	–5.89	–2.57	<0.001
SB_{ilr1}	4.16	–2.13	10.46	0.194	7.29	3.15	11.44	0.001	9.13	7.41	10.85	<0.001
LPA_{ilr1}	5.03	–0.32	10.37	0.065	1.58	–2.06	5.23	0.394	–0.36	–1.72	0.99	0.597
$MVPA_{ilr1}$	–1.59	–4.08	0.89	0.208	–7.32	–9.62	–5.02	<0.001	–4.53	–5.33	–3.74	<0.001
SES (Medium)	–0.11	–2.62	2.39	0.929	0.87	–2.68	4.41	0.632	–1.73	–2.55	–0.92	<0.001
SES (High)	0.38	–1.96	2.72	0.748	–1.53	–5.14	2.07	0.405	–3.34	–4.22	–2.46	<0.001
Sex	–0.30	–1.89	1.30	0.716	–7.51	–9.17	–5.85	<0.001	–8.47	–9.16	–7.78	<0.001
Age	2.38	1.71	3.05	<0.001	0.39	–0.16	0.93	0.163	–0.24	–0.31	–0.17	<0.001
Study ID (random)	2.44	0.56	10.73	<0.001	NA	NA	NA	NA	0.55	0.08	3.58	0.108

CI – confidence interval, ID – identification number, *ilr*1 – isometric log-ratio (the first coordinate), LPA – light-intensity physical activity, MVPA – moderate-to-vigorous physical activity, NA – not applicable, SB – sedentary behavior, SES – socioeconomic status.

The final model aggregates individual models, each containing a complete set of pivot coordinates (i.e., *ilr*1, *ilr*2, and *ilr*3). Four individual models were built, rearranging the component order to express each behavior as *ilr*1. The final model combines the first pivot coordinates (*ilr*1) from these individual models.

Marginal R^2 represents the variance explained by the fixed effects, as calculated using the equation by Nakagawa and Schielzeth [5].

Conditional R^2 represents the variance explained by the entire model, including both fixed and random effects, as calculated using the equation by Nakagawa and Schielzeth [5].

[a] Model was tested using the dataset from which participants with missing socioeconomic status were excluded.

6 References

1. Templ M, Kowarik A, Filzmoser P. Iterative stepwise regression imputation using standard and robust methods. Comput Stat Data Anal 2011; **55**: 2793-806. https://doi.org/10.1016/j.csda.2011.04.012
2. Rubin DB. Multiple imputation for nonresponse in surveys. Hoboken, NJ: Wiley-Interscience; 2004.
3. Allen LN, Wigley S, Holmer H, Barlow P. Non-communicable disease policy implementation from 2014 to 2021: a repeated cross-sectional analysis of global policy data for 194 countries. Lancet Glob Health 2023; **11**: e525-e33. https://doi.org/10.1016/S2214-109X(23)00042-6
4. Mansournia MA, Nazemipour M. Recommendations for accurate reporting in medical research statistics. The Lancet 2024; **403**: 611-12. https://doi.org/10.1016/S0140-6736(24)00139-9
5. Nakagawa S, Schielzeth H. A general and simple method for obtaining R2 from generalized linear mixed-effects models. Methods in Ecology and Evolution 2013; **4**: 133-42. https://doi.org/10.1111/j.2041-210x.2012.00261.x
